# Rapid behavioral screening in the planarian *Dugesia japonica* is a biologically relevant system to study neurotoxicity of organophosphorus pesticides mixtures

**DOI:** 10.3389/ftox.2026.1753546

**Published:** 2026-03-26

**Authors:** Danielle Ireland, Rui Wang, S. Grace Fuselier, Christina Rabeler, Rebecca T. Somach, Rudy J. Richardson, Eva-Maria S. Collins

**Affiliations:** 1 Department of Biology, Swarthmore College, Swarthmore, PA, United States; 2 Department of Environmental Health Sciences, University of Michigan, Ann Arbor, MI, United States; 3 Department of Neurology, University of Michigan, Ann Arbor, MI, United States; 4 Center for Computational Medicine & Bioinformatics, University of Michigan, Ann Arbor, MI, United States; 5 Michigan Institute for Computational Discovery & Engineering, University of Michigan, Ann Arbor, MI, United States; 6 Michigan Institute for Data & AI in Society, University of Michigan, Ann Arbor, MI, United States; 7 Department of Physics and Astronomy, Swarthmore College, Swarthmore, PA, United States; 8 Department of Neuroscience, Perelman School of Medicine, University of Pennsylvania, Philadelphia, PA, United States; 9 Center of Excellence in Environmental Toxicology, University of Pennsylvania, Philadelphia, PA, United States

**Keywords:** chemical interactions, developmental neurotoxicity, loewe additivity, new approach method, potentiation, synergism

## Abstract

**Introduction:**

Organophosphorus pesticides (OPs) are an economically important and chemically diverse class of compounds that share acetylcholinesterase (AChE) inhibition as their mode of action for acute toxicity. Chronic OP exposure at doses below significant AChE inhibition has been shown to cause neurotoxicity and developmental neurotoxicity in mammals, implying a role for other molecular targets. Because the large number of OPs are often used in mixtures and many require bioactivation, high throughput screening (HTS) methods with human-relevant xenobiotic metabolism are necessary to study how OPs interact and exert their toxic effects.

**Methods:**

We screened 21 binary mixtures of 7 OPs (acephate, chlorpyrifos (CPF), diazinon, dichlorvos (DDVP), malathion, parathion, and profenofos) in equimolar ratios ranging from 0.1 to 10 µM in adult and regenerating *Dugesia japonica* planarians, which have metabolic competence for OPs. Using an automated screening platform with 22 readouts, we quantified effects on lethality, stickiness, and behavior on days 7 and 12 post-exposure. We compared experimental outcomes to predictions from a Loewe concentration addition model to evaluate whether the adverse outcomes of binary mixtures of OPs can be predicted based on their shared mode of action. To contextualize the behavioral results, we also tested four OP mixtures for AChE inhibition at day 12 post-exposure in regenerating planarians.

**Results:**

The tested OP binary mixtures showed additive and non-additive interactions, including potentiation, synergism and antagonism, that differed across mixtures, readouts, and developmental stage (adult vs. regenerating planarians). Regarding AChE inhibition, CPF-parathion and CPF-malathion mixtures acted additively, while diazinon-DDVP showed antagonism, and diazinon-profenofos showed synergism. Some behavioral outcomes, such as stickiness, correlated with effects on AChE inhibition while others did not. Adult and regenerating planarians differed in the extent of non-additive OP interactions.

**Discussion:**

The direct comparison of effects of OP mixtures on the adult vs. the developing nervous system allowed us to identify greater synergistic effects in regenerating planarians compared to adults, emphasizing the need to better understand the effects of OPs on the developing brain. Due to the conservation of key enzymes in OP toxicokinetics/toxicodynamics between planarians and humans, lessons learned from the planarian system are likely to be relevant to human health.

## 1 Introduction

Organophosphorus pesticides (OPs) are a prevalent class of insecticides that have been in use in the United States for >70 years, starting with parathion (PT) in 1948 (International Agency for Research on Cancer, 2017), followed by malathion (MAL) and diazinon (DZN) in the 1950s (Agency for Toxic Substances and Disease Registry ATSDR, 2003; National Pesticide Information Center, 2009). OPs are cost-effective and provide protection against a variety of pests (Metcalfe, 2002). They kill pests through inhibition of the enzyme acetylcholinesterase (AChE) (Mileson et al., 1998; Russom et al., 2014; Costa, 2018; Taylor, 2018), which is key to regulating cholinergic signaling and highly conserved between species (Wiesner et al., 2007). Thus, OPs are also toxic to humans, and safety concerns have led to both tighter regulations and declined usage of these pesticides in recent years. According to a report from the U.S. Environmental Protection Agency from 2017, OP use declined from approximately 70 million pounds to 20 million pounds between 2000 and 2012 (Atwood and Paisley-Jones, 2017). PT was banned in the U.S. in 2003 (International Agency for Research on Cancer, 2017), and other OPs have been increasingly replaced by other pesticides (Grube, 2011), which may or may not be safer alternatives.

Despite phasing-out and replacement initiatives, many OPs are still in regular use in the U.S., the exact number of which can be difficult to determine. According to a 2003 publication by the Environmental Protection Agency, there were approximately 40 OPs registered for use (Woodruff, 2003). The widespread usage of OPs across a broad range of agricultural products (e.g., almonds/walnuts, alfalfa, broccoli and other fruits and vegetables) (Metcalfe, 2002) can lead to exposure to multiple OPs simultaneously or consecutively for both agricultural workers and end consumers (Barr et al., 2004; Leonel Javeres et al., 2020). The default approach to predict mixture toxicity has relied on concentration addition models (U.S. EPA, 2003). However, concerns exist that such multi-OP exposures could lead to increased toxicity that cannot be predicted *via* simple addition from knowledge of the toxicity of individual OPs. The possibility of enhanced toxicity for OP co-exposures was first noticed with MAL within a few years of the first OPs arriving on the market. Many OPs, including MAL, require bioactivation into their active oxon or other functional metabolites to exert their toxic effects (Amitai et al., 1998; Buratti et al., 2003). MAL alone has low toxicity to humans because it is rapidly detoxified by the liver enzyme carboxylesterase, preventing accumulation of its toxic metabolite, malaoxon (MALO) (Schopfer et al., 2025). However, other OPs inhibit carboxylesterase and thus co-exposure of MAL with other OPs can cause increased toxicity. In “Silent Spring” (1962), Rachel Carson wrote: “*(…) when malathion and certain other organic phosphates are administered simultaneously a massive poisoning results–up to 50 times as severe as would be predicted on the basis of adding together the toxicities of the two.”* Her statement is based on studies that demonstrated that PT and other compounds that inhibit carboxylesterase potentiated MAL’s ability to inhibit acetylcholinesterase when co-administered (Murphy and Dubois, 1957; Murphy et al., 1959). Additional studies have since emerged that demonstrate potentiation and synergism between OPs and between OPs and other chemicals. For example, the oxon of chlorpyrifos (CPF) also inhibits carboxylesterase (Buratti and Testai, 2005) and prior exposure to CPF oxon (CPO) therefore augments MAL toxicity in mice (Jansen et al., 2009; Cole et al., 2010; Costa, 2018). More-than-additive toxicity in regard to AChE inhibition and neurobehavioral responses was also observed when adult rats were exposed to a mixture of CPF and three other OPs (including acephate (ACE) and DZN), in the absence of MAL (Moser et al., 2005). The greater-than-additive toxicity of the OP mixture was most prevalent at lower OP concentrations, which did not significantly inhibit brain AChE when each chemical was administered alone (Moser et al., 2005).

However, because mammalian studies have focused on only a few OP mixtures (primarily CPF + MAL, CPF + DZN), our knowledge about the toxicity of OP mixtures remains limited. This lack of data on OP mixtures is due to the tremendous time and financial cost of mammalian toxicity testing (Meigs, 2018). There is strong advocacy in the field of toxicology to replace vertebrate tests with new approach methods (NAMs), primarily *in vitro* tests in human cells or tissues, to increase throughput and human relevance, as evidenced by the recent strategic plans of major U.S. regulatory agencies (ICCVAM Interagency Coordinating Committee on the Validation of Alternative Methods, 2018; USEPA, 2021; US Federal Drug Administration, 2025). While multiple studies on individual OPs have been conducted in induced pluripotent stem cell derived human neural stem cells (e.g. (Yamada et al., 2017; Di Consiglio et al., 2020)), few cell culture studies, derived from either human or mammalian cells, have evaluated OP mixtures. Existing studies have primarily used lymphocytes or hepatocellular carcinoma cells with limited capability for OP bioactivation (Qu et al., 2021) and have focused on cytotoxic and genotoxic effects (Ojha and Srivastava, 2014; Sultana Shaik et al., 2016; Barrón Cuenca et al., 2022). Thus, there is a data gap for the effect of OP mixtures on neuronal cells and nervous system function, a limitation shared across mixture studies (Martin et al., 2021).

As the examples above demonstrate, xenobiotic metabolism is key for understanding the toxic profile and interactions of OPs. Many *in vitro* methods have been shown to be poor predictors of OP toxicity due to lack of metabolic machinery (Aylward and Hays, 2011; Silva et al., 2015). Some newer methods have been able to incorporate some aspects of OP xenobiotic metabolism (Wu et al., 2017; Di Consiglio et al., 2020), though these have not yet been used in mixture studies. In addition, systems employing whole organism non-mammalian models will become increasingly important for closing knowledge gaps and complementing *in vitro* and computational studies. To the best of our knowledge, only one OP mixture study has been reported in an organismal NAM, namely in *Caenorhabditis elegans*, using four different OPs, including CPF (Wang et al., 2021), to study effects on locomotion. It was found that the type of OP interaction was concentration-dependent, with some OP mixtures displaying synergistic effects at low concentrations and additive effects at higher concentrations while other mixtures showed the reverse (Wang et al., 2021).

The freshwater planarian *Dugesia japonica* is a well-suited invertebrate model for studying OP neurotoxicity and developmental neurotoxicity [reviewed in Ireland and Collins (2023); Collins et al. (2024)]. The planarian nervous system consists of about 2000–10,000 neurons depending on worm size (Brown et al., 2018), is compartmentalized into distinct neuronal subpopulations and contains almost all neurotransmitters found in humans (Cebrià et al., 2002; Ross et al., 2017; Ireland and Collins, 2023). Following transection, planarians regenerate all missing structures, including the central nervous system through many of the same key events as mammalian neurodevelopment (Ross et al., 2017; Ireland and Collins, 2023), allowing neuroregeneration to be used as a model for neurodevelopment. Planarians possess key features that make them amenable to rapid screening [reviewed in Ireland and Collins (2023); Collins et al. (2024)]: They are aquatic, a few mm long, have a fast developmental timeline compared to mammalian models, and their behavior can be used as a quantitative readout of brain function. Planarian phenotypic profiling can detect neurotoxic chemicals (Zhang et al., 2019a; Zhang et al., 2019b; Ireland et al., 2022a). Most importantly for the context of studying OP toxicity, *D. japonica* has two cholinesterases (DjChEs), which are considered evolutionary ancestors of human AChE (Hagstrom et al., 2017, 2018). DjChEs are inhibited by OPs and carbamates (Hagstrom et al., 2017, 2018), and OP-inhibited DjChEs can be reactivated by oximes (Hagstrom et al., 2017) like human cholinesterases. Moreover, we showed that *D. japonica* can bioactivate CPF and DZN throughout neurodevelopment (Ireland et al., 2022a). *D. japonica* also contains paraoxonase-like and carboxylesterase activities (Ireland et al., 2022a), which are important for OP detoxification in mammals (Srikanth and Seth, 1990; Poet et al., 2003; Costa et al., 2008; Alejo-González et al., 2018).

**FIGURE 1:**
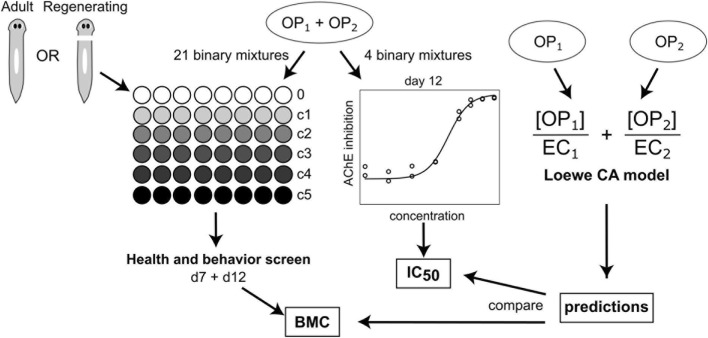
Experimental overview. Adult or regenerating planarians exposed to one of 21 binary OP mixtures were screened for effects on health and behavior at days 7 and 12 (d7 and d12, respectively) post-exposure. For each mixture and readout, a benchmark concentration (BMC) was derived. A subset of 4 OPs were also studied for their effects on AChE inhibition in regenerating planarians at day 12 post-exposure and to calculate the concentration that induced 50% inhibition (IC_50_). The BMC and IC_50_ values were compared to those predicted by a Loewe concentration addition (CA) model for the various effective concentrations (EC, either BMC or IC_50_) to determine if the effects could be explained by an additive model based on shared mode of action.

We have previously individually studied 7 OPs (ACE, CPF, DZN, dichlorvos (DDVP), MAL, PT, and profenofos (PFS)) in *D. japonica* planarians (Ireland et al., 2022b) and found that the different OPs have varying toxicity phenotypes, suggesting differences in toxicokinetics or differential effects on multiple targets. Building on these data, here we screened 21 binary mixtures of these 7 OPs (Figure 1). We performed behavioral screening on adult and regenerating planarians using at least 5 concentrations up to 10 µM for all 21 mixtures and measured AChE inhibition for 4/21 mixtures in regenerating planarians. We used our previously published behavioral data on individual OPs (Ireland et al., 2022b) to determine whether a Loewe concentration addition (CA) model, which assumes a shared mode of action, could describe the experimental OP mixture data. The CA model is widely used in mixture toxicity assessment (Berenbaum, 1985; Cedergreen, 2014) and is the default model used for regulatory assessment of OP mixtures (Office of Pesticide Programs, U.S. Environmental Protection Agency EPA, 2000; U.S. EPA, 2003). Based on existing knowledge of OP interactions and our previous findings from the single OP screen in planarians (Ireland et al., 2022b), we hypothesized that some OPs would act additively for some readouts but not others and that some non-additive effects may be independent of effects on AChE inhibition. Our results show that binary interactions between these OPs can be non-additive, depending on the mixture, readout and developmental stage of the planarian (adult vs. regenerating). Because key enzymes in OP toxicokinetics and toxicodynamics are conserved between planarians and humans, the insights gained here are likely to be relevant to human health and will require further investigation in targeted mechanistic studies in human cells.

## 2 Materials and methods

### 2.1 Test system

Asexual *Dugesia japonica* freshwater planarians were used for all experiments. This lab culture was originally obtained from Shanghai, China and has been maintained in the laboratory for over a decade. Planarians were kept in planarian water (Ireland et al., 2025a) in BPA-free polypropene plastic containers at 20 °C in a Panasonic refrigerated incubator in the dark. Feeding occurred 1x/week using either organic beef liver (from a local farm) or organic chicken liver (Bell & Evans, Fredericksburg, PA). Cleaning was performed 2×/week according to standard protocols (Dunkel et al., 2011). Intact worms fasted for at least 5 days that moved normally in the container were manually and arbitrarily selected for experiments. Worm length was estimated from image analysis of the screening data to range between 4 and 9 mm across all conditions. To induce regeneration/development, planarians were transected in the region between the auricles and the pharynx with an ethanol-sterilized razor blade. Chemical exposure began within 3 h of amputation. Larger worms (6-11 mm) were used for transection, so that regenerating planarians were of similar size as intact/adult planarians for screening.

### 2.2 Chemical information and binary mixture preparation

Chemicals used are listed in Table 1. Chemical stock solutions were prepared in 100% dimethyl sulfoxide (DMSO, Sigma-Aldrich, Saint Louis, MO) and stored at −20 °C. Except for MAL and PFS (Chem Service, West Chester, PA), all chemicals were purchased from Sigma-Aldrich. The identity and purity of the OPs was verified using high performance liquid chromatography/mass spectrometry with a UV detector (Lotus Separations, Princeton, NJ). The exact procedures and results from the mass spectrometry analysis are provided in Supplementary Data Sheet 1.

**TABLE 1:** Chemicals. LogP information listed is according to PubChem. The purity listed was provided by the vendor and independently confirmed using mass spectrometry (Lotus Separations, Princeton, NJ).

Chemical name	Abbreviation	CAS	DTXSID	log*P*	Molecular weight	Vendor purity (%)	Verified purity (%)
Acephate	ACE	30560-19-1	DTXSID8023846	−0.8	183.17	98	N/A
Chlorpyrifos	CPF	2921-88-2	DTXSID4020458	5.0	350.59	100	99.5
Diazinon	DZN	333-41-5	DTXSID9020407	3.8	304.35	98	99.5
Dichlorvos	DDVP	62-73-7	DTXSID5020449	1.4	220.98	98	≥99.9%
Malathion	MAL	121-75-5	DTXSID4020791	2.4	330.38	98.6	≥99.9%
Parathion	PT	56-38-2	DTXSID7021100	3.8	291.26	100	88.8%
Profenofos	PFS	41198-08-7	DTXSID3032464	4.7	373.6	97	98.3%

Each of the 21 OP binary mixtures were tested at 1:1 M ratios with the final concentration of each OP ranging from 0.1 to 10 µM in semi-log steps. This concentration range was chosen to span the mid-range of concentrations previously tested in single exposure (Ireland et al., 2022a). Some mixtures were rescreened at one to two additional lower concentrations (in semi-log steps) as the original range did not capture the no-observed-effect-level (Supplementary Table S1). Note, MAL, ACE, and PFS are racemic mixtures (Wang et al., 2006) but were treated as one chemical entity, ignoring possible differences between enantiomers, to simplify analysis. The mixtures were prepared by first generating 400X stock solutions in 100% DMSO of each OP. Stock solutions of two OPs were then mixed to create 1:1 mixtures of two OPs at 200X of the highest test concentration. This stock solution was then serially diluted in DMSO to create 200X stock solutions of each test concentration. 200X stock plates were prepared ahead of time and stored at −20 °C until the day of plate set-up (described in Section 2.3). On the day of plate set-up, the 200X stock plates were thawed and diluted to 10X in planarian water and then subsequently added to the screening plates to obtain the final 1X nominal test concentrations. In all test concentrations, the final concentration of DMSO was 0.5% (v/v). We have previously shown that this concentration of DMSO does not have effects on adult or regenerating *D. japonica* in our screening paradigm (Hagstrom et al., 2015; Ireland et al., 2020).

### 2.3 High-throughput screening (HTS) and benchmark concentrations (BMCs)

HTS was conducted as previously described (Zhang et al., 2019a; Ireland et al., 2020; Ireland et al., 2022b). In brief, one adult or regenerating/developing planarian was loaded in 200 µL of solution per well of a tissue culture-treated 48-well plate (Genesee Scientific, San Diego, CA). Each plate contained five chemical concentrations (one per row, with *n* = 8 planarians) and one in-plate 0.5% (v/v) DMSO (solvent; *n* = 8) control. Following chemical addition, each plate was sealed with a ThermalSeal RTS seal (Excel Scientific, Victorville, CA) (Zhang et al., 2019a) to minimize evaporation and cross-contamination. Chemical solutions were not replaced and planarians were fasted for the duration of the experiment.

For each mixture, three plates filled with adult planarians and three filled with regenerating tails were screened, for a total of *n* = 24 per concentration and worm type. Plate orientation was shifted in the replicates to control for possible edge effects (Zhang et al., 2019a). Positional effects may arise for several reasons: For some assays, the stimulus is administered from the outside, thus planarians in the outer wells may be exposed to a stronger stimulus than those in the interior wells. Another factor is increased optical aberrations at the outer wells compared to the center wells. Planarians may see the shadows of their conspecifics through the transparent plastic of the multi-well plate which may affect their motion; specimens on the outside have fewer neighbors than interiorly located planarians. Finally, the plate is sealed with a plastic film and the edges of the sealing may not be as strong as the center, thus creating variable conditions across the plate. Between screening, we stored the plates in the dark at room temperature (20 °C–22 °C).

Screening was performed on days 7 and 12 of exposure using an automated platform consisting of a commercial robotic microplate handler (Hudson Robotics, Springfield Township, NJ) and multiple computer-controlled cameras and assay stations as described in detail in Zhang et al. (2019a), Ireland et al. (2020), Ireland et al. (2022b), Fuselier et al. (2023). Four types of assays were performed: 1) phototaxis/locomotion/lethality, 2) stickiness, 3) thermotaxis, and 4) noxious heat sensing/scrunching and the readouts listed in Table 2 were quantified. Phototaxis was assayed as in Ireland et al. (2022b) by imaging under different lighting conditions: 1-min red light (first dark cycle), 1-min green light (light cycle), 2-min red light (second dark cycle), 1-min blue light (light cycle). Center of mass tracking was used to quantify spatial exploration (wall preference) and the average speed and amount of time resting in different light cycles (Table 2). The speed and resting readouts were modified from the original single OP screen (Ireland et al., 2022b) to combine readouts across the same light period to increase robustness and decrease noise (Table 2). Identification of lethality was performed manually by a reviewer who was blind to the chemical identities. The stickiness assay was performed by shaking the plate at a fixed rotation per minute and manually quantifying the number of planarians that remain stuck to the well bottom (Ireland et al., 2020). Thermotaxis quantified the amount of time the planarian stayed in a cool region of the well as previously described (Zhang et al., 2019a). The noxious heat sensing assay gradually warmed the plate using a peltier and the rate and strength of the reaction (Ireland et al., 2020) and the ability of the planarian to execute the expected scrunching (Cochet-Escartin et al., 2015) behavior were quantified as in Zhang et al. (2019a), Ireland et al. (2020). The phototaxis and stickiness assays were tested on day 7. All assays were tested on day 12.

**TABLE 2:** Readouts and their associated benchmark response (BMR). For a more detailed description of these measures, see Zhang et al. (2019a), Ireland et al. (2020), Ireland et al. (2022b), Fuselier et al. (2023). If a readout can be assessed in both the positive (+) and negative (−) directions, the directionality of the BMR is listed in parentheses.

Readout	Brief description	Adult BMR	Regenerating BMR
Lethality	% dead	d7: 10 d12: 20	d7: 10 d12: 15
Stickiness	% stuck individuals	d7: 50 d12: 50	d7: 50 d12: 50
Speed (dark)^a^	Mean speed (mm/s) in 2nd dark cycle	d7: 50/50 (+/−) d12: 40/50 (+/−)	d7: 35/30 (+/−) d12: 45/45 (+/−)
Resting (dark)^a^	Fraction of time spent resting in 2nd dark cycle	d7: 65/40 (+/−) d12: 45/55 (+/−)	d7: 35/60 (+/−) d12: 50/55 (+/−)
Speed (blue)^a^	Mean speed (mm/s) in blue cycle	d7: 55/50 (+/−) d12: 45/50 (+/−)	d7: 40/40 (+/−) d12: 70/40 (+/−)
Resting (blue)^a^	Fraction of time spent resting in blue cycle	d7: 35/30 (+/−) d12: 30/40 (+/−)	d7: 35/35 (+/−) d12: 40/40 (+/−)
Wall preference	Fraction of time spent in outer region of well	d7: 35 (−) d12: 25 (−)	d7: 40 (−) d12: 35 (−)
Locomotor bursts (total)	Sum of locomotor bursts in phototaxis assay	d7: 11/7 (+/−) d12: 11/6 (+/−)	d7: 18/11 (+/−) d12: 9/6 (+/−)
Phototaxis	Average speed in blue cycle - 2nd minute of 2nd dark cycle	d7: 35/45 (+/−) d12: 30/40 (+/−)	d7: 30/20 (+/−) d12: 35/35 (+/−)
Thermotaxis	Fraction of time in cold zone	d12: 45 (−)	d12: 40 (−)
Scrunching	% planarians that did not scrunch in response to noxious heat	d12: 25	d12: 50
Noxious stimuli (rate)	Rate of change in displacement in response to heat	d12: 35/25 (+/−)	d12: 35/30 (+/−)
Noxious stimuli (strength)	Median displacement at end of noxious heat	d12: 50/65 (+/−)	d12: 50/65 (+/−)

^a^ The readout was modified from the original OP screen (Ireland et al., 2022b). BMRs were recalculated based on the single OP data.

Raw imaging data were analyzed blinded without chemical information in MATLAB (MathWorks, Natick, MA) and Python using previously described scripts (Zhang et al., 2019a; Ireland et al., 2020; Ireland et al., 2022b). Benchmark concentrations (BMCs) were calculated for every readout and chemical to quantify potency using the Rcurvep R package (Hsieh et al., 2019) as described previously for the individual OPs (Ireland et al., 2022b) in R (4.5.0 (R Core Team, 2021)). Regenerating and adult planarians and day 7 and day 12 were treated separately. The data were processed as described in Ireland et al. (2022b) and the benchmark responses (BMRs) from the single OP screen were used to calculate the BMC for each readout to allow for direct comparison of single OP and mixture data (Table 2). Some readouts had been added or modified from the original single OP screen (Table 2). For these, we recalculated the new readouts for the single OP data and used the resulting BMRs from the single OP data only. For all readouts, we report the median BMCs calculated from bootstrapped results (*n* = 1,000 iterations). The lower and upper limits (5th and 95th percentiles, respectively) and hit confidence scores of the BMC for each readout and binary mixture are listed in Supplementary Data Sheet 2 and for the individual OPs in Supplementary Data Sheet 3. For readouts that can be affected in both directions (e.g., speed), BMRs and BMCs were calculated for each direction (Table 2). For some readouts, the confidence intervals spanned most or the entire tested concentration range, which is due to limitations with how the Rcurvep BMC pipeline handles non-monotonic concentration-response curves (Hsieh et al., 2019; Pacis et al., 2025).

### 2.4 *In vivo* AChE activity assays

We previously quantified AChE activity in adult planarians following 12 days of exposure to the single OPs (Ireland et al., 2022b). This time point was chosen to give insight into the steady state inhibition levels seen following long-term exposure to identify correlations between AChE inhibition levels and behavioral outcomes. Because our focus was on studying developmental neurotoxicity of OP binary mixtures, we performed AChE activity (Ellman et al., 1961) assays on regenerating planarians exposed to the individual OPs at varying concentrations for 12 days following the same methodology as previously described for adult planarians in Ireland et al. (2022b) to complement that existing data set. Some additional single OP data points were also tested in adult planarians to bolster the original complete dose response curves (Ireland et al., 2022b). We also exposed regenerating planarians to select concentrations of some binary OP mixtures and quantified their AChE activity at day 12. All chemicals and concentration ranges tested are listed in Supplementary Table S2.

In brief, 36 planarians (intact for adult data and pre-pharyngeally transected for regenerating data) were exposed to a specific concentration of a single OP or binary mixture in 0.5% (v/v) DMSO or to 0.5% DMSO (v/v) (solvent control). Planarians were bulk exposed (6 worms/1.2 mL solution) in tissue culture-treated 12-well plates (Genesee Scientific), using a ratio of planarian/volume (1/200 µL) in each well consistent with HTS. Plates were sealed and stored in the dark for 12 days. We excluded worms that divided or worms from wells with death. Post-exposure, planarians were washed 3X with planarian water and homogenized in 200 µL 1% (v/v) Triton X-100 in PBS as described in Hagstrom et al. (2017), Hagstrom et al. (2018). An Ellman assay (Ellman et al., 1961) was performed using an AChE Activity Assay kit (Sigma-Aldrich) as previously described (Ireland et al., 2022b; Fuselier et al., 2023). Protein concentration was measured using a Coomassie (Bradford) protein assay kit (Thermo Scientific, Waltham, MA) and used to normalize AChE activity. In each experiment, normalized AChE activity was compared to the average respective solvent control samples (set at 100% activity) tested on the same day. Activity measurements were performed with three technical replicates per condition. The inhibition dose response curves were fit to a log-logistic equation (setting the lower limit to 0, the upper limit to 100, and using the IC_50_ (concentration resulting in 50% inhibition), and Hill slopes as parameters) with the drc R package (Ritz et al., 2015).

### 2.5 *In vitro* AChE activity assays

To examine the immediate interaction of OPs with DjChE, we performed *in vitro* Ellman assays on planarian homogenates. Twenty to thirty intact *D. japonica* that were fasted for 5–9 days were homogenized in 200 µL 1% (v/v) Triton X-100 in PBS as described above. Following homogenization, protein concentration was measured using a Coomassie (Bradford) protein assay kit (Thermo Scientific, Waltham, MA). The planarian homogenate was diluted to 1 μg/μL protein in 1% (v/v) Triton X-100. We have previously shown that CPF and DZN are unable to inhibit DjChE in *in vitro* homogenates, suggesting bioactivation *via* desulfuration by cytochrome P450s is absent in these preparations (Ireland et al., 2022a). Thus, we tested the oxon metabolites chlorpyrifos oxon (CPO, ChemService, CAS# 5598-15-2, 98% purity, MET-11459B) and malaoxon (MALO, Sigma-Aldrich (Supelco), CAS# 1634-78-2, ≥98.2% purity (NMR by supplier), which were prepared in 100% DMSO at 200x concentration of the final dilution. Preliminary analysis of the single oxon data showed ∼4 orders of magnitude difference in potency (IC_50_). Thus, we prepared the mixture at a constant ratio of 0.007 ([CPO]/[MALO]) based on the preliminary IC_50_ values (CPO: 1 μM, MALO: 13.6 µM) and spanned ranges from 1/16th to 8× the IC_50_ values (Supplementary Table S3). The mixture was prepared from 400X stocks in 100% DMSO. The chemicals were then diluted to 10X the final concentration in 1% (v/v) Triton X-100. 36 μL of diluted planarian homogenate was added to 4 µL of 10x chemical stock, vortexed and incubated for 10 min at room temperature. After 10 min, the homogenate-chemical mixture was transferred to a 96-well plate in triplicate and AChE activity was quantified using an AChE assay kit (Abcam, Cambridge, United Kingdom) as previously described. All assays had a 0.5% (v/v) DMSO control prepared alongside the other samples that was used for normalization and set to 100% activity. The same homogenate was used for all samples on the same day. Experiments were tested in duplicate using fresh independent homogenate. Data processing and analysis were the same as with the *in vivo* assays described in Section 2.4.

### 2.6 Mixture modeling

#### 2.6.1 Loewe concentration addition (CA) model for predicting binary mixture BMCs

The median BMCs (with confidence intervals) of the single OP data for both adult and regenerating planarians individually (Supplementary Data Sheet 3) were used to calculate the predicted binary equimolar mixture BMC using the Loewe CA model (Loewe, 1928), implemented in R (4.5.0 (R Core Team, 2021)). The CA model assumes that two chemicals share the same mode of action, which in the case of OPs is AChE inhibition, the current paradigm by which OP usage is regulated in the U.S. (Mileson et al., 1998; Office of Pesticide Programs, U.S. Environmental Protection Agency (EPA), 2021). This CA model is also what is generally used by regulatory agencies for mixture safety assessment (U.S. EPA, 2003; Kortenkamp et al., 2009). According to the CA model, the following relationship holds (Equation 1):

$$\frac{C_{1}}{{BMC}_{1}}+\frac{C_{2}}{{BMC}_{2}}=1$$ (1)

*C*_1_ and *C*_2_ are the concentrations of the individual OPs. BMC_1,2_ are the respective BMCs for a given readout, i.e., the predicted concentration for which the response exceeds the BMR for that readout. BMCs for the individual OPs were multiplied by 10^6^ to convert to µM concentrations as input. At equimolar ratios and reporting the mixture concentration as per component concentration (*C*_1_ = *C*_2_ = *C*_mix_), we can determine the BMC of the mixture (as per component concentration, with $\left.{BMC}_{mix}total=2*\;{BMC}_{mix}\right)$ as:

$${BMC}_{mix}=\frac{1}{\left(\;\frac{1}{{BMC}_{1}}+\frac{1}{{BMC}_{2}}\right)}$$ (2)

For each readout and each binary mixture, the relative potency factor (RPF) of chemical 2 was calculated relative to chemical 1, according to: RPF_2_ = BMC_1_/BMC_2_. Using the RPF, Equation 2 simplifies to Equation 3 which was used to calculate the predicted mixture BMCs:

$${BMC}_{mix}=\frac{{BMC}_{1}}{\left(1+{RPF}_{2}\right)}$$ (3)

If one of the two OPs did not have an active BMC for a given readout, its BMC was considered infinite and the BMC_mix_ was predicted to equal the BMC of the active OP (Liu et al., 2015). We then compared the predicted mixture BMCs to the experimentally observed BMCs for each readout (Supplementary Figures S1–S4). To allow for fair comparisons with the observed BMCs, which would have a maximum of 10 μM, only predicted mixture BMCs of up to 10 µM were considered. BMCs with overlapping confidence intervals for a given readout and mixture were considered additive. Observed BMCs less than the predicted BMCs represent synergistic effects, while observed BMCs greater than predicted BMCs represent antagonistic relationships. If only one of the predicted or observed mixtures had a BMC, the confidence intervals were considered non-overlapping.

#### 2.6.2 Loewe concentration addition (CA) model for predicting AChE IC_50_

As AChE inhibition is a shared mechanism for the OPs studied here, it is reasonable to assume that the OPs would act additively regarding AChE inhibition. To test this, we produced four binary equimolar 1:1 mixtures (CPF.PT, CPF.MAL, DDVP.DZN, and DZN.PFS) and determined their effect on AChE activity (see Section 2.4). The tested mixture concentrations are listed in Supplementary Table S2. We plotted the experimental data for these four binary mixtures in MATLAB (R2024b, MathWorks) and fit the inhibition concentration-response curves to a log-logistic equation, with the lower limit set to 0, upper limit to 100, and using the IC_50_ as a fit parameter. To calculate the predicted IC_50_ for the mixture using the CA model, we used the single compound IC_50_ values and confidence intervals as inputs into Equation 3, wherein the IC_50_s were used in place of the BMCs and RPF_2_ was calculated as RPF_2_ = IC_50(1)_/IC_50(2)_. To generate the predicted mixture curves, we also determined other effect levels from the individual curves (IC_10_, IC_30_, etc.) and used those as inputs for Equation 3. The same strategy was used to model the *in vitro* data (Section 2.5) but the ratios of CPO to MALO were taken into account as follows (Equation 4):

$${IC50}_{mix}total=\frac{1}{\left(\;\frac{f1}{{IC50}_{1}}+\frac{f2}{{IC50}_{2}}\right)}$$ (4)

Where *f*_1_, *f*_2_ designate the fraction of compounds 1 and 2 in the total mixture. While this approach assumes identical lower limits and slopes, it can be applied to calculation of the IC_50_ even with varying slopes as the results only differ slightly (Ritz et al., 2021).

Additionally, for the *in vitro* data, we also compared the observed percent AChE inhibition to the expected concentration additive model using the method previously described in Richardson et al. (2001). Briefly, log-logit regression lines were fit to the data from the individual OPs. Next, for describing OP concentrations in the mixture, concentrations of CPO were converted into equivalent concentrations of MALO, i.e., the concentration of MALO that would lead to the same logit inhibition at the tested concentration of CPO. The concentration in the mixture was therefore determined as [CPO_MAO-equivalent_] + [MALO] and the logit expected percent inhibition was modeled as

$$Y=Y_{o\left(MALO\right)}+b_{MALO}X$$

Where Y_o(MALO)_ is the Y intercept and b_MALO_ is the slope of the log-logit regression line for MALO. X is log of the concentration of the mixture described above. The linear regression of the observed *versus* expected logit percent inhibition was calculated and the resulting slope was compared to the expected slope of 1.0 based on the assumption of concentration additivity. To determine whether the obtained slope was significantly different from 1.0, we calculated the median response for each concentration of the actual mixture and applied a coefficient-based hypothesis test (coeftest) in MATLAB.

## 3 Results

### 3.1 Binary OP mixtures cause distinct behavioral profiles

**FIGURE 2:**
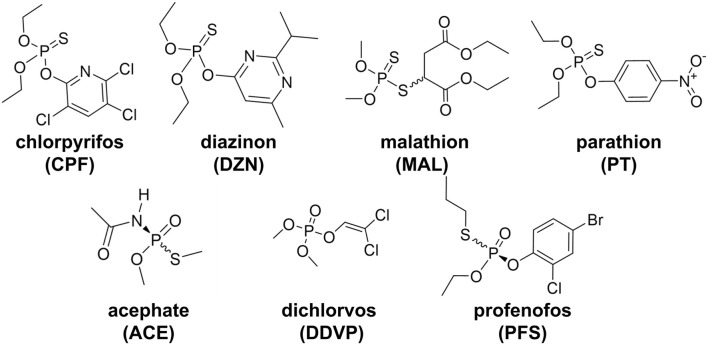
Chemical structures of the seven OPs. For the three structures with a chiral center, wavy single bonds indicate that the racemic mixtures were used in this study and were evaluated as one chemical entity.

We previously found that 12 days exposure to 7 individual OPs - ACE, DZN, DDVP, MAL, PT, and PFS (Figure 2) - produced different behavioral phenotypes that could not be explained solely by levels of AChE inhibition (Ireland et al., 2022b). Five of these OPs (CPF, DZN, DDVP, PFS, and MAL) produced distinct behavioral defects in regenerating planarians compared to adult planarians, suggesting that they affect different targets during neurodevelopment (Ireland et al., 2022b).

Four of these OPs are phosphorothioates (CPF, PT, DZN, MAL) requiring bioactivation *via* cytochrome P450 enzymes into their active oxon metabolite to inhibit AChE (Buratti et al., 2003, 2005). DDVP does not require bioactivation and can inhibit AChE directly (Assis et al., 2007; Hagstrom et al., 2017). ACE, MAL, and PFS are chiral compounds and commercial products are racemic mixtures of their respective S- and R- enantiomers. While little is known about possible differences in toxicity for the ACE enantiomers, it has been shown that MAL enantiomers result in distinct metabolic profiles in human hepatocellular carcinoma cells (Yan et al., 2019). For PFS, the two enantiomers have been shown to differ in potency and their ability to directly inhibit AChE *in vitro* (French et al., 1985; Nillos et al., 2007). Moreover, AChE from different species has been found to show enantiomer selectivity - while the R-(+)-enantiomer of PFS was found to be more potent *in vitro* against AChE of several species, the reverse was true *in vivo*, highlighting the complexity of the situation (Nillos et al., 2007). Because no enantiomer selectivity studies have been conducted yet in planarians, we treated these OPs as single chemical entities for this study. ACE requires deacetylation to form its active metabolite, methamidophos (Kao and Fukuto, 1977). As the only hydrophilic compound among these seven OPs, ACE was found to be the least potent of these OPs in *D. japonica* planarians as it caused few effects and did not significantly inhibit DjChE in adult planarians up to the maximum tested concentration of 316 µM (Ireland et al., 2022b).

**FIGURE 3:**
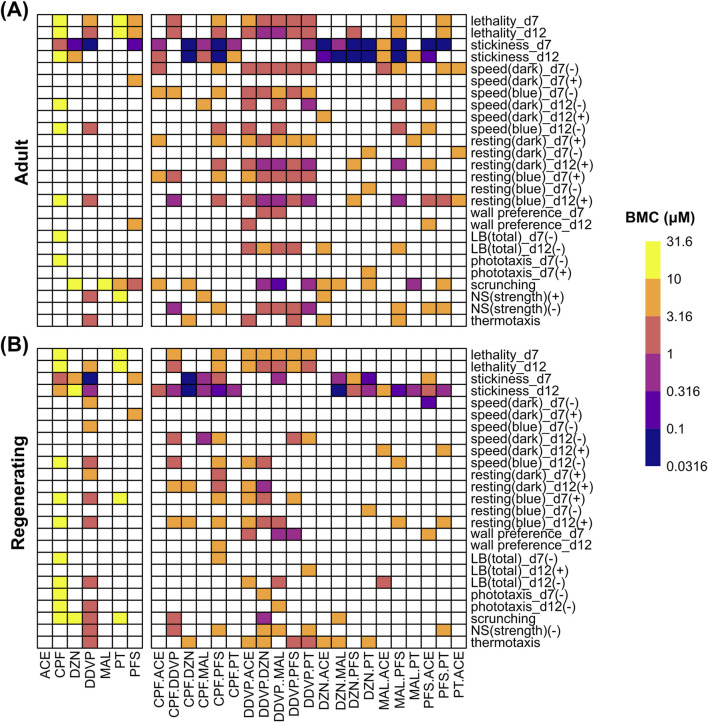
Comparison of OP mixture toxicity *versus* that of individual OPs. Heatmaps comparing the benchmark concentrations (BMCs) for the single OPs [data from Ireland et al. (2022a)], capped at 31.6 µM, and the binary OP mixtures, tested up to 10 μM, in adult **(A)** and regenerating **(B)** planarians. For readouts that can have effects in both directions, the BMCs are separated by either the positive (+) or negative (−) direction. LB, locomotor bursts; NS, noxious stimuli. For each worm type, only readouts/directions that are active in at least one condition are shown.

Here, we tested 21 equimolar binary mixtures of these 7 OPs. Adult and regenerating planarians (*n* = 24) were exposed for 12 days to at least 5 concentrations each of the 21 mixtures, testing 1:1 combinations of the 7 OPs up to a maximum concentration of 10 µM. To allow for comparisons with our original individual OP exposures (Ireland et al., 2022b), we report all concentrations as the concentrations of the individual component OPs. Thus, if the mixture concentration is listed at 10 μM, this means that each OP was used at 10 µM. Figure 3 summarizes the toxicity profiles of the 7 individual OPs [data from Ireland et al. (2022b)] and the 21 binary mixtures studied here. The toxicity profiles varied greatly across the different mixtures and across planarian developmental stages (adult vs. regenerating). Generally, adult planarians were more sensitive to exposure to the OP mixtures as the BMCs were generally lower. In adult planarians, stickiness was the most sensitive endpoint as almost all DZN- and many PFS-containing mixtures had increased stickiness at BMCs between 0.0316 and 0.1 µM. Although DDVP alone showed great potency in stickiness at day 7 (BMCs of ∼0.1 µM in both adult and regenerating planarians), all mixtures containing DDVP, including DDVP.DZN, did not have any significant effects on stickiness. Mixtures containing DDVP affected more readouts in adults than other mixtures, while in regenerating planarians there was less of a distinction. While ACE alone did not cause any effects in our previous screen at equivalent test concentrations (the minimum BMC was 229 µM (Ireland et al., 2022b)), combining ACE with other OPs led to increased toxicity in adult planarians, suggesting potentiation, evidenced by more hits at the same or lower concentrations than what was observed for exposure to the other OP. This trend was generally not observed in regenerating planarians. A notable exception was MAL: the MAL.ACE mixture showed hits in more readouts than MAL alone in both adult and regenerating planarians. MAL also showed more than additive effects in combination with other OPs, as in both adult and regenerating planarians more hits were detected in the MAL-containing mixtures than what was observed for the individual OPs. To formally evaluate the mixtures, we compared the observed BMCs of the mixtures to BMCs predicted from the Loewe CA model (Methods Section 2.6).

### 3.2 Experimental OP mixture effects *versus* predicted mixture effects

**FIGURE 4:**
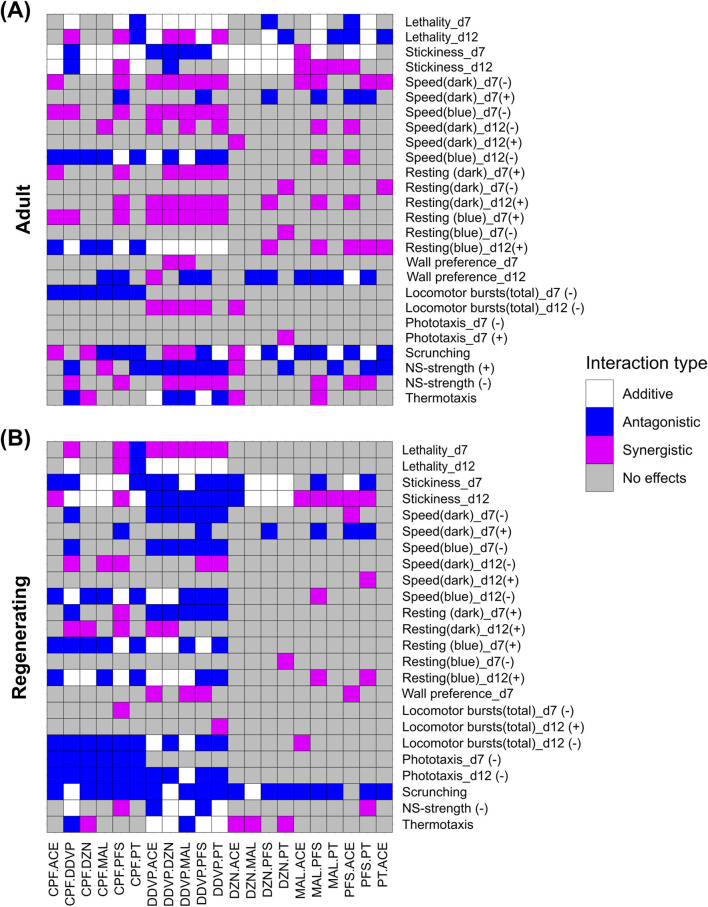
Heatmap showing whether observed adverse outcomes match additive modeling. BMCs and their corresponding confidence intervals were compared for each readout and mixture across the experimental and CA model predicted data (Supplementary Figures S1–S4) in adult **(A)** and regenerating **(B)** planarians. Each readout was then classified according to their interaction type: “Additive” indicates the confidence intervals overlapped, “Antagonistic” indicates the predicted confidence intervals were below that of the experimental data, and “Synergistic” indicates the experimental BMC confidence intervals were below the predicted confidence intervals. No effects refer to cases where both the experimental and predicted data do not show effects.

The CA model assumes that the studied compounds share the same mode of action, which in the case of OPs is AChE inhibition (Mileson et al., 1998; Office of Pesticide Programs, U.S. Environmental Protection Agency (EPA), 2000). However, if some of the effects we observed in the behavioral screening were due to mechanisms other than AChE inhibition or if one OP affects the toxicokinetics or toxicodynamics of another OP, it is possible that OP interactions would deviate from the predictions of an additive model. Thus, we sought to determine how well the CA model could predict behavioral outcomes of the binary OP mixtures. Figure 4 summarizes the results from the comparison of the experimentally observed mixture data to the predicted data in a heatmap; in Supplementary Figures S1–S4 we show all comparisons of model and experiment per readout. While many of the readouts showed additive behavior (white boxes in Figure 4), consistent with the idea that OPs share their mode of action, there were also multiple cases of non-additive behavior (colored boxes). For adult planarians, we observed more synergistic effects whereas for regenerating planarians more of the non-additive effects were antagonistic. Several of the non-additive effects were chemical specific. For example, in both adult and regenerating planarians, several MAL containing mixtures (MAL.ACE, MAL.PFS, and MAL.PT) showed synergistic effects in stickiness at day 12. Mixtures containing DDVP or CPF showed more non-additive effects than other mixtures across readouts. Whether those effects were synergistic or antagonistic depended on the readout, the other OP in the mixture, and the developmental state of the planarian. For example, in adult planarians, some CPF-containing mixtures (CPF.MAL, CPF.PFS, and CPF.PT) showed antagonistic effects for scrunching, whereas CPF.ACE and CPF.DZN showed synergism. In regenerating planarians, all CPF containing mixtures showed antagonistic effects on scrunching, with the exception of CPF.DDVP which was additive. Notably, CPF.PFS and CPF.DDVP caused non-additive effects in more than half of all readouts, particularly in regenerating planarians, showing both synergism and antagonism, depending on the readout. Mixtures containing DDVP had very different interaction patterns between adult and regenerating planarians. In regenerating planarians, most DDVP-containing mixtures had many readouts with antagonistic interactions, except for day 7 lethality, which was synergistic in all DDVP mixtures. In contrast, in adult planarians, most readouts from DDVP-containing mixtures showed synergistic behavior, with the exception of stickiness at day 7, NS-strength (−), and thermotaxis, which showed primarily antagonism. In addition to the per readout comparisons, we also compared the minimum BMC (BMC_min_) per mixture across all readouts, by calculating the ratio of log_10_(BMC_min(predicted)_/BMC_min(experimental)_) to understand if the overall toxicity of the experimental mixtures deviated meaningfully from the Loewe CA predictions. We found that discrepancies between experimental and predicted BMC_min_ were generally small for adult planarians (<1), whereas in regenerating planarians, many mixtures showed experimental potency greater than 10X the predicted concentration (log_10_ ratio >1) (Table 3).

**TABLE 3:** Comparison of minimum BMC (BMC_min_) across any readout for each mixture in the experimental or CA model prediction. BMC_min_ is in µM. Ratio is log_10_(BMC_min(predicted)_/BMC_min(experimental)_). NA indicates no active BMCs were calculated in this mixture.

Mixture	Adult			Regenerating		
	Experimental BMC_min_	Predicted BMC_min_	Ratio	Experimental BMC_min_	Predicted BMC_min_	Ratio
CPF.ACE	0.92	1.69	0.27	2.15	1.70	−0.10
CPF.DDVP	0.54	0.09	−0.79	1.00	0.09	−1.03
CPF.DZN	0.07	0.19	0.43	0.10	1.44	1.16
CPF.MAL	1.00	1.69	0.23	0.68	1.70	0.40
CPF.PFS	0.07	0.25	0.54	0.24	1.24	0.72
CPF.PT	0.71	1.69	0.37	0.92	1.70	0.27
DDVP.ACE	1.44	0.09	−1.20	1.59	0.10	−1.20
DDVP.DZN	0.47	0.06	−0.87	0.95	0.10	−0.98
DDVP.MAL	0.32	0.09	−0.54	0.74	0.10	−0.87
DDVP.PFS	1.37	0.07	−1.29	0.99	0.10	−1.01
DDVP.PT	0.46	0.09	−0.70	1.61	0.10	−1.21
DZN.ACE	0.09	0.22	0.37	6.22	9.15	0.17
DZN.MAL	0.08	0.22	0.44	0.09	9.15	2.00
DZN.PFS	0.07	0.12	0.24	3.00	3.04	0.01
DZN.PT	0.07	0.22	0.48	0.27	8.01	1.48
MAL.ACE	2.72	13.50	0.70	1.47	45.29	1.49
MAL.PFS	0.07	0.29	0.62	0.21	4.56	1.34
MAL.PT	0.91	3.63	0.60	0.68	12.97	1.28
PFS.ACE	0.08	0.29	0.56	0.23	4.56	1.31
PFS.PT	0.08	0.29	0.59	0.79	4.56	0.76
PT.ACE	7.91	4.96	−0.20	NA	NA	NA

### 3.3 Role of AChE inhibition

We previously showed that in adult planarians toxicity resulting from individual exposure to the seven OPs was not correlated with AChE inhibition (Ireland et al., 2022b). To complement these data, we measured AChE activity on day 12 in regenerating planarians and quantified the AChE IC_50_ (Table 4; Supplementary Figure S5). As with the adults, ACE did not cause significant AChE inhibition in regenerating planarians at the highest soluble concentration of 316 µM (data not shown). CPF, MAL, PFS and PT had comparable AChE IC_50_ values across adult and regenerating planarians, within standard error measures. DZN was slightly more potent in regenerating planarians, while DDVP showed ×19 greater potency in regenerating planarians compared to adult planarians.

**TABLE 4:** Day 12 AChE IC_50_ (µM) of regenerating and adult planarians. Raw data and fitted curves are shown in Supplementary Figure S5. SE, standard error.

OP	Regenerating IC_50_ (SE)	Adult IC_50_ (SE)^a^
ACE	>316	>316
CPF	0.27 (0.041)	0.18 (0.043)
DDVP	0.0050 (0.0010)	0.095 (0.012)
DZN	0.056 (0.008)	0.16 (0.06)
MT	4.9 (2.0)	4.7 (1.1)
PFS	0.057 (0.016)	0.044 (0.012)
PT	0.21 (0.038)	0.16 (0.02)

^a^ Based on original data (Ireland et al., 2022b), expanded with additional experiments.

To test whether effects on AChE inhibition were additive as would be expected for these OPs, we selected 4 of the 21 binary mixtures (CPF.PT, CPF.MAL, DDVP.DZN, and DZN.PFS) to experimentally determine their effects on AChE inhibition in regenerating planarians after 12 days of static exposure. All of the tested OPs except for ACE, which poorly inhibits DjChE (Table 4), were included in at least one of these mixtures. Two OPs (CPF and DZN) were included in two mixtures each, so we could assess how changing one OP would affect the AChE inhibition profile. CPF.PT was chosen because this combination has previously been found to show different inhibition profiles depending on the sequence of *in vivo* administration in rats, suggesting interactions of the two OPs (Karanth et al., 2001). Since CPF inhibits carboxylesterase, the main detoxifying enzyme for MAL (Buratti and Testai, 2005; Crow et al., 2012), and CPF.MAL mixtures have been reported to have synergistic effects on salmon AChE (Laetz et al., 2009), CPF.MAL was also studied. These two mixtures consist only of OPs that need bioactivation to their active oxon metabolites *via* cytochrome P450-mediated oxidative desulfuration to inhibit AChE (Buratti et al., 2003, 2005). Thus, we also studied a mixture that included one OP that can inhibit directly AChE (DDVP) and one that must be bioactivated (DZN), which would likely lead to differences in inhibition kinetics. The last mixture was a binary mixture of PFS and DZN, which had similar IC_50_ values in regenerating planarians (Table 4).

**FIGURE 5:**
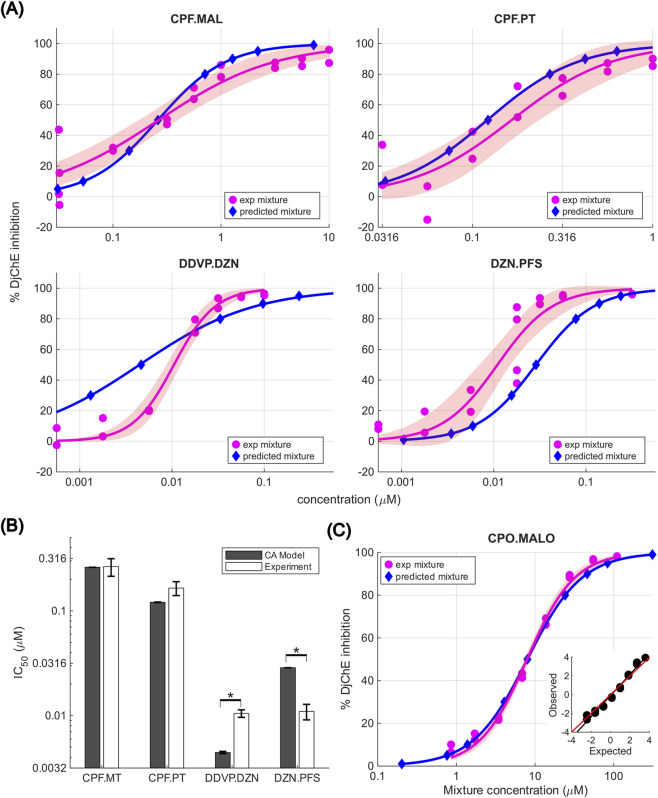
Comparison of the AChE inhibition curves for OP mixtures and their predicted values. **(A)** Comparison of the concentration response curves of the selected equimolar binary OP mixtures showing the experimental data and associated Hill fit, including 95% confidence interval bands, compared to the response predicted by the CA model in regenerating planarians after 12 days of exposure. **(B)** Bar plot comparing the day 12 IC_50_ from the CA model and experimental results for all 4 tested binary mixtures in regenerating planarians. Error bars represent SE. * indicates *p* < 0.05, Student’s t-test. In **(A)** and **(B)**, mixture concentrations are reported as per component concentrations. **(C)** Comparison of the *in vitro* CPO.MALO concentration response curve after 10-min treatment showing the experimental data and associated Hill fit, including 95% confidence interval bands, compared to the response predicted by the CA model. Concentrations shown are total mixture concentrations. Inset: Regression of expected logit percent inhibition vs. observed logit percent inhibition (black) vs. expected slope = 1.0 (red). No significant difference was found between these slopes (See Methods Section 2.6.2). Dose response curves for individual oxon exposure are provided in Supplementary Figure S7.

These 4 OP mixtures were tested at 1:1 equimolar ratios across a range of concentrations to obtain concentration response curves (Supplementary Figure S6). Using the fit parameters from the individual OP curves in regenerating planarians (Supplementary Figure 5A), we calculated the predicted day 12 IC_50_ for the 4 binary mixtures using the CA model and compared that to experimentally determined IC_50_ values for these mixtures (Figures 5A,B). CPF.PT and CPF.MAL showed additive effects on DjChE inhibition. In contrast, DDVP.DZN showed an antagonistic relationship wherein the experimental IC_50_ was higher (less potent) than that predicted by the CA model and DZN.PFS showed synergistic behavior wherein the experimental IC_50_ was lower (more potent) than that predicted by the CA model (Figure 5B). For DDVP.DZN, the slopes of the predicted *versus* the experimentally observed inhibition curves were also very different from each other (Figure 5A). The mixture showed less than additive effects at low concentrations (antagonism) and more than additive effects at high concentrations (synergism).

Because we only observed additive effects for CPF.MAL in the 12-day static exposure experiments and it is possible that compensatory mechanisms may change the interactions over the long incubation times, we also performed *in vitro* acute inhibition experiments using the oxon metabolites of these compounds and found only additive interactions at the tested concentrations following 10 min treatment (Figure 5C; Supplementary Figure S7).

## 4 Discussion

### 4.1 Behavioral phenotypes induced by OP mixtures suggest non-additive effects

We previously studied the effect of 7 individual OPs (ACE, CPF, DDVP, DZN, MAL, PT and PFS) and showed that they had distinct toxicity profiles that differed between adult and regenerating planarians and that could not be explained by the levels of AChE inhibition alone (Ireland et al., 2022b). This suggested that the OPs have different targets in addition to their shared target AChE or have differing toxicokinetics. These data imply that the toxicity of OP mixtures likely cannot simply be predicted by an additive model based on a shared target, as is currently done for OP risk assessments (Office of Pesticide Programs, U.S. Environmental Protection Agency (EPA), 2000). Taking advantage of the rapid screening capacity of the planarian system, we therefore analyzed the 21 possible binary OP mixtures of these 7 OPs to compare experimental data to the predictions of an additive model to stress test this regulatory assumption.

Exposure to the OP mixtures caused many behavioral effects, generally at lower concentrations than with the individual OPs alone. Stickiness was a frequently affected and sensitive endpoint, similar to what we observed for the individual OPs (Ireland et al., 2022b). We have previously shown that increased stickiness (the ability of worms to adhere to a substrate while being shaken at a fixed rotational frequency (Ireland et al., 2020)) is correlated with the shared effects of OPs on cholinergic signaling, leading to increased mucus secretion (Hagstrom et al., 2018; Ireland et al., 2022b). Acute cholinergic toxicity in humans is also characterized by increased secretions (Pope et al., 2005; Peter et al., 2014; Taylor, 2018). Interestingly, while exposure to the individual OPs primarily caused hits in increased stickiness at day 7 but not day 12, more hits in day 12 stickiness were observed in the OP mixtures than were seen in the individual OPs. Altered toxicokinetics or toxicodynamics when multiple OPs are present simultaneously may explain these temporal differences. While DDVP alone caused effects in stickiness on day 7 for adults and day 7 and 12 for regenerating planarians at low concentrations, all but 2 of the DDVP-containing mixtures (DDVP.PT for adults and DDVP.MAL for regenerating planarians) did not show increased stickiness and thus showed antagonistic interactions at these readouts. Neither PT nor MAL individually caused increased day 7 stickiness, suggesting that interactions between the OPs cause these differences in stickiness phenotypes for DDVP-containing mixtures.

We have previously demonstrated that noxious heat sensation is attenuated when DjChE activity is reduced, either chemically or *via* RNA interference (Hagstrom et al., 2018). Adult planarians exposed individually to either DDVP or PT showed increased NS (strength) scores, indicative of a weaker response to noxious heat than controls. In contrast, while none of the OPs caused reduced NS (strength) scores, indicative of a stronger response to noxious heat than controls, in adult planarians, nine OP binary mixtures showed effects demonstrating synergistic interactions at this readout. Many of these mixtures contained DDVP, which seemed to switch directionality in this readout when combined with other OPs. Thus, in adult planarians, all DDVP-containing mixtures showed patterns consisting of antagonistic effects on NS-strength (+) and synergistic effects on NS-strength (−). This trend was not observed in regenerating planarians, where DDVP-mixtures had primarily additive effects on NS-strength (−), except for DDVP.ACE and DDVP.PFS which showed antagonistic behavior. Additionally, while only DDVP affected thermotaxis when testing individual OPs, several of the mixtures not containing DDVP (CPF.DZN, DZN.ACE, DZN.MAL, DZN.PT in regenerating planarians and CPF.DZN, DZN.ACE, and MAL.PT in adult planarians) caused bioactivity in thermotaxis demonstrating synergistic interactions. Similar trends were seen in other readouts, where some mixtures showed more effects and at lower concentrations than what would be expected from concentration addition. Together, our data imply that one cannot simply deduce mixture behavior of one OP binary mixture from another, and that differences in both toxicokinetics and toxicodynamics need to be considered to understand mixture effects.

### 4.2 AChE inhibition can explain some behavioral effects in the OP mixtures

To contextualize the behavioral data and investigate its relationship with AChE inhibition, we evaluated AChE inhibition at the end of the 12 days exposure. We have previously published the day 12 AChE IC_50_ values for the individual OPs using adult planarians (Ireland et al., 2022b). Here, we report the day 12 IC_50_ values for regenerating planarians. We generally observed similar inhibition and ranking of the OPs, with the exception of DDVP, which was found to be about twenty times more potent in inhibiting AChE in regenerating planarians than in adults. For four selected OP mixtures, we used the data from the individual OPs to predict inhibition curves using the Loewe CA model, which assumes a shared mode of action. Two mixtures (CPF.PT and CPF.MAL) showed additive effects whereas the other two tested mixtures (DDVP.DZN and DZN.PFS) showed antagonistic and synergistic effects, respectively.

In humans, exposure to MAL leads to relatively little toxicity because it is rapidly detoxified by carboxylesterase before it can be converted to its active oxon form (MALO) (Buratti and Testai, 2005; Schopfer et al., 2025). However, many compounds, including the oxon metabolite of CPF CPO, inhibit carboxylesterase (Buratti and Testai, 2005). It has been shown that prior exposure to CPO enhances MAL toxicity *in vivo* in mice (Jansen et al., 2009; Cole et al., 2010; Costa, 2018). Similarly, synergistic effects on AChE inhibition were also found in salmon co-exposed to CPF and MAL (Laetz et al., 2009). Synergism was also found with a mixture of CPF + MAL in *in vitro* studies of pure housefly AChE in the absence of any metabolic confounders, including carboxylesterase (Arora and Kumar, 2015). Thus, we were surprised to find only additive effects on AChE inhibition in the CPF.MAL mixture in regenerating planarians. While we have shown that *D. japonica* exhibits carboxylesterase activity (Ireland et al., 2022a), one or more carboxylesterase gene(s) remain to be identified.

It is possible that CPO cannot inhibit the planarian carboxylesterase(s) or alternatively that they are not required for MAL detoxification, which will require further investigation. Alternatively, by measuring AChE activity only on day 12 of exposure, we may be missing the effects of those interactions and future studies should explore these earlier dynamics. Because it is unclear what time window would be the appropriate window to measure, we utilized acute *in vitro* experiments to evaluate the interaction of the corresponding metabolites (CPO and MALO), thus not requiring bioactivation. We found that, after a 10-min exposure, those mixtures behaved additively, like the *in vivo* mixtures of the parent compounds after day 12 exposure. While the planarian homogenates used for these experiments likely contain carboxylesterase activity, this remains to be verified. Additionally, MALO can also directly inhibit carboxylesterase (Main and Dauterman, 1967). Thus, the interplay of reactions is likely complex and will require further investigation to understand the temporal dynamics of CPO and MALO inhibition of carboxylesterase versus detoxification of MALO and which reactions would dominate within a planarian homogenate at the time scale used (10 min). While the effects on AChE inhibition were additive, CPF.MAL and CPF.PT primarily showed antagonism on individual behavioral readouts in regenerating planarians. However, the differences between CA model predictions and experimental data were small, with the experimental BMC_min_ at 0.68 µM compared to the predicted BMC_min_ at 1.70 µM for CPF.MAL and 0.92 µM (experimental) *versus* 1.70 µM (predicted) for CPF.PT, suggesting that an additive model works well for these binary mixtures.

In addition to the DDVP.DZN mixture showing an overall antagonistic interaction on day 12 AChE IC_50,_ the slope of the experimental curve was much steeper than of the predicted curve, showing little inhibition at the lowest test concentrations, followed by a rapid increase that exceeds the predicted inhibition at high concentrations. These differences suggest antagonistic interactions at low OP concentrations and synergistic interactions at high concentrations. In support of potential antagonism at low concentrations, the predicted BMC_min_ (0.1 µM) for DDVP.DZN in regenerating planarians was nearly 10-fold lower than that of the experimental mixture (0.95 µM). The predicted BMC_min_ was derived from effects on day 7 stickiness, which were absent in the experimental mixture. In fact, all DDVP-containing mixtures showed no hits in stickiness, despite DDVP alone having strong effects on increased stickiness at low concentrations (BMC of ∼0.1 µM). As discussed above, increased stickiness is correlated with AChE inhibition and increased cholinergic signaling (Hagstrom et al., 2018; Ireland et al., 2022b). Thus, there may be a potential connection here between specific behavioral readouts and changes in AChE inhibition. In contrast, the experimental BMC_min_ at higher concentrations (0.95 µM) was derived from effects on scrunching, which showed additive interactions. These results emphasize that the nature of the interaction can differ depending on the concentrations of the OPs in the mixture. Consequently, concentration is an important variable to consider when studying OP interactions. Of course, studies requiring multiple concentration levels of each compound in a mixture greatly enlarges the scale of the investigation, but this problem can be managed by employing HTS approaches.

### 4.3 OP mixtures showed stronger synergistic effects in regenerating than in adult planarians

Our behavioral and AChE inhibition data show non-additive interactions of the tested binary OP mixtures. This is important information to unravel the molecular targets and pathways these compounds affect during neurodevelopment, which can be refined in future mechanistic studies. However, an important question is whether the extent of the non-additive effects is sufficiently large to be biologically meaningful and outside of expected inter-experiment variability (Martin et al., 2021). Thus, it has been recommended that regulatory evaluation of mixture effects should focus on integrated measures of overall toxicity, such as the potency of the most sensitive endpoint (BMC_min_) (Kortenkamp et al., 2009). When comparing the BMC_min_ per mixture across all readouts (Table 3), we found that discrepancies between experimental and predicted BMC_min_ were generally small for adult planarians. In contrast, in regenerating planarians, many mixtures showed experimental potency greater than 10× the predicted concentration. DZN.MAL in regenerating planarians showed the greatest discrepancy between experimental and CA predicted values, with a 100-fold lower experimental BMC_min_ than predicted. These differences indicate that in the developing planarian nervous system, mechanisms other than AChE inhibition may be the primary contributors to OP neurotoxicity and that knowledge of the OP mixture effects on adult planarians is insufficient to predict effects on regenerating planarians.

Importantly, this difference in experimental versus predicted values specifically in regenerating planarians was observed, despite adult planarians being generally more sensitive to the individual OPs and their mixtures. The increased sensitivity in adult planarians is not specific to OPs and has been observed with a variety of different chemicals (Zhang et al., 2019a; Ireland et al., 2022b; Ireland et al., 2025b). While this is initially counterintuitive given that one would expect the developing organism to be more vulnerable, it is possible this is due to differences in basal metabolism and/or toxicokinetics. While oxygen consumption is similar between intact and regenerating planarians, glycolysis is increased in regenerating planarians (Osuma et al., 2018). Additionally, regenerating worms secrete mucus for wound protection (Peiris et al., 2014), which may impede chemical uptake. Lastly, it is possible that the act of regeneration allows these specimens to overcome some types of toxicity as development is a dynamic process where repair and compensatory mechanisms are highly prevalent. Further research will be needed to better understand these potential toxicokinetic/toxicodynamic differences between adult and regenerating planarians to contextualize the observed differences in adverse outcomes and distinguish these from effects on neurodevelopmental-specific targets or key events.

### 4.4 Limitations and opportunities

There are several limitations from this study that need to be considered when interpreting the results. The binary mixtures studied here are simplifications that are useful for directing future mechanistic studies but do not capture realistic human exposure scenarios, in terms of both number of components and their relative contribution, nor do they take into account chirality. The nominal concentrations tested here have not been matched to environmentally relevant concentrations and any extrapolations to human exposure would require more knowledge on important toxicokinetic parameters in planarians. All chemical exposures presented here were static and are expressed in terms of the original nominal concentration. OPs are a highly unstable chemical class and can be subject to hydrolysis in aqueous solutions and adsorption into plastic due to their lipophilicity (Di Consiglio et al., 2020); thus, it is possible that the concentrations changed over the course of the 12-day exposure. Importantly, the level of stability in aqueous cultures depends on both the specific OP and the composition of the media (Bian et al., 2020). It is also unknown whether any interactions between the two OPs (or their degradation/metabolic byproducts) could affect OP stability in the binary mixtures or if the degradation/metabolic byproducts contributed to any adverse effects. Because the tested OPs (both alone and in the mixtures) caused significant AChE inhibition when measured at day 12, that would suggest that the OPs were still present at sufficiently high concentrations at the end of the 12-day exposure period. Future studies examining the actual OP concentrations in both the media and within the planarians will be needed to unravel these possibilities. While we studied only simultaneous exposures, future studies should also include sequential OP exposures, as human exposures to OPs involve both. It has been shown in adult male rats that simultaneous exposure to PT and CPF is more similar in toxicity profile to sequential exposure with CPF first and PT second, whereas exposure to PT followed by CPF is less toxic (Karanth et al., 2001). This difference was attributed to the amount of carboxylesterase inhibition by the two OPs (Karanth et al., 2001). As planarians also have carboxylesterase activity (Ireland et al., 2022a), similar studies can be conducted in adult and developing organisms in parallel at a much larger scale than is possible in mammals.

The use of historical data for the single OPs in the CA model introduces inter-experiment variability as a confounding factor for interpretation of the results. Different chemical batches were used for the single OP *versus* the mixture screen and the lack of a controlled planarian diet (Pacis et al., 2025) introduces biological variability. Being mindful of this variability and the fact that behavioral readouts are intrinsically noisy (Zhang et al., 2017), we took a conservative approach in determining non-additive effects by only considering non-overlapping confidence intervals (as used in determination of the BMCs) between experimental and predicted data as evidence for non-additive effects. For readouts with bioactivity in only either the predicted or experimental data, it is important to acknowledge that inter-screen variability in per-readout potency can be significant, depending on the compound and length of time separation between screens (Pacis et al., 2025) and thus could contribute to these differences. However, changes in overall potency considering all readouts (BMC_min_) were found to be small in a previous study of inter-screen variability (Pacis et al., 2025) and overall potency (BMC_min_) was largely conserved in the CA predictions in adult planarians while significant synergism was observed in regenerating planarians. Thus, because the chemical stocks and treatments were consistent within the mixture screen, the differences that we observed between adult and regenerating planarians treated with the same mixtures, as well as the OP-mixture specific differences in behaviors, cannot be explained by interexperimental variability.

The knowledge gained here on the differences in OP interactions between adult and developing organisms, and that other molecular targets besides AChE likely contribute to OP toxicity, will be important for understanding other types of mixture compositions, where interactions between these other targets may play a larger role. OPs are often combined or used with other types of pesticides, e.g., carbamates or pyrethroids, to increase their effectiveness (Martin et al., 2003; Khan et al., 2013; Madgwick and Kanitz, 2023), and some studies have shown non-additive effects for OP-carbamate and OP-pyrethroid mixtures (Arora et al., 2017). Moreover, adjuvants, such as piperonyl butoxide, are frequently added to enhance carbamate or pyrethroid toxicity by inhibiting CYP450 enzymes, which are the primary detoxification enzymes in insects (Buratti et al., 2003). Because of this property, piperonyl butoxide has been suggested as a useful tool to study the individual contributions of OPs to mixture toxicity as it reduces the toxicity of OPs that require bioactivation (Ankley et al., 1991). The use of piperonyl butoxide can also be used to distinguish the relative contributions of the two enantiomers of PFS to *in vivo* inhibition of AChE, as the S-enantiomer has been shown to inhibit AChE 34X better following bioactivation in *in vitro* mouse liver microsomes (Wing et al., 1983). Lastly, having a HTS-compatible system with metabolic competence allows for direct comparison of environmentally relevant mixtures with per-component mixtures, to dissect how different chemicals affect mixture toxicity and thus indicate which combinations of chemicals pose the greatest health hazard. Such complex mixture studies have been carried out in developing zebrafish (Geier et al., 2018) and human liver spheroids (Addicks et al., 2023) for per-and polyfluoroalkyl substances and could be similarly employed for pesticides in planarians.

In conclusion, our study is, to the best of our knowledge, the first NAM study of this scope on OP mixtures, examining 21 binary OP mixtures in adult and developing organisms with metabolic competence, thus providing a wealth of new information that can be used to guide mechanistic studies of OP toxicity. While the use of an additive model was sufficient to capture the toxicity profiles in adult planarians, it would be non-protective for regenerating planarians. The low cost and relatively high throughput of planarian behavioral HTS allows for future large-scale comparative studies of different exposure scenarios using environmentally relevant mixtures.

## Data Availability

The original contributions presented in the study are included in the article/Supplementary Material, further inquiries can be directed to the corresponding author.

## Supplementary material

SUPPLEMENTARY DATA SHEET 1 Mass spectroscopy results to independently confirm chemical purity. .zip files containing reports from Lotus Separations details methods and results of high performance liquid chromatography / mass spectroscopy analysis.

SUPPLEMENTARY DATA SHEET 2 Benchmark concentration (BMC), confidence intervals and hit scores for all readouts in adult and regenerating planarians exposed to OP mixtures. The BMC is listed as BMC_median. BMC_cil and BMC_ciu represent the lower and upper confidence intervals, respectively. Hit_confidence scores greater than 0.5 were considered hits. Some readouts were assessed in both increasing (+) and decreasing (−) directions and are listed separately. Concentrations are listed in log(M).

SUPPLEMENTARY DATA SHEET 3 Benchmark concentration (BMC), confidence intervals and hit scores for all readouts in adult and regenerating planarians exposed to individual OPs. The BMC is listed as BMC_median. BMC_cil and BMC_ciu represent the lower and upper confidence intervals, respectively. Hit_confidence scores greater than 0.5 were considered hits. Some readouts were assessed in both increating (+) and decreasing (−) directions and are listed separately. Concentrations are listed in log(M). Some readouts were previously reported in Ireland et al. (2022a).

SUPPLEMENTARY DATA SHEET 4 Supplementary Figures and Tables.

## References

[B1] Addicks G. C. Rowan-Carroll A. Reardon A. J. F. Leingartner K. Williams A. Meier M. J. (2023). Per- and polyfluoroalkyl substances (PFAS) in mixtures show additive effects on transcriptomic points of departure in human liver spheroids. Toxicol. Sci. An Official J. Soc. Toxicol. 194, 38–52. 10.1093/toxsci/kfad044 37195416 PMC10306399

[B2] Agency for Toxic Substances and Disease Registry (ATSDR) (2003). Toxicology profile for malathion. Atlanta, GA: U.S. Department of Health and Human Services, Public Health Service.

[B3] Alejo-González K. Hanson-Viana E. Vazquez-Duhalt R. (2018). Enzymatic detoxification of organophosphorus pesticides and related toxicants. J. Pesticide Sci. 43, 1–9. 10.1584/JPESTICS.D17-078 30363124 PMC6140661

[B4] Amitai G. Moorad D. Adani R. Doctor B. P. (1998). Inhibition of acetylcholinesterase and butyrylcholinesterase by chlorpyrifos-oxon. Biochem. Pharmacol. 56, 293–299. 10.1016/s0006-2952(98)00035-5 9744565

[B5] Ankley G. T. Dierkes J. R. Jensen D. A. Peterson G. S. (1991). Piperonyl butoxide as a tool in aquatic toxicological research with organophosphate insecticides. Ecotoxicol. Environ. Saf. 21, 266–274. 10.1016/0147-6513(91)90065-W 1868783

[B6] Arora S. Kumar A. (2015). Binary combinations of organophosphorus pesticides exhibit differential toxicity under oxidised and un-oxidised conditions. Ecotoxicol. Environ. Saf. 115, 93–100. 10.1016/j.ecoenv.2015.01.003 25682586

[B7] Arora S. Balotra S. Pandey G. Kumar A. (2017). Binary combinations of organophosphorus and synthetic pyrethroids are more potent acetylcholinesterase inhibitors than organophosphorus and carbamate mixtures: an in vitro assessment. Toxicol. Lett. 268, 8–16. 10.1016/j.toxlet.2016.12.009 27988393

[B8] Assis C. R. D. Amaral I. P. G. Castro P. F. Carvalho L. B. J. Bezerra R. S. (2007). Effect of dichlorvos on the acetylcholinesterase from tambaqui (*Colossoma macropomum*) brain. Environ. Toxicol. Chem. 26, 1451–1453. 10.1897/06-488R1.1 17665685

[B9] Atwood D. Paisley-Jones C. (2017). Pesticides industry sales and usage 2008 - 2012 market estimates. Washington, DC: U.S. Environmental Protection Agency.

[B10] Aylward L. L. Hays S. M. (2011). Consideration of dosimetry in evaluation of ToxCast^TM^ data. J. Appl. Toxicol. 31, 741–751. 10.1002/jat.1626 21381051

[B11] Barr D. B. Bravo R. Weerasekera G. Caltabiano L. M. Whitehead R. D. Olsson A. O. (2004). Concentrations of dialkyl phosphate metabolites of organophosphorus pesticides in the U.S. population. Environ. Health Perspect. 112, 186–200. 10.1289/ehp.6503 14754573 PMC1241828

[B12] Barrón Cuenca J. de Oliveira Galvão M. F. Ünlü Endirlik B. Tirado N. Dreij K. (2022). *In* vitro cytotoxicity and genotoxicity of single and combined pesticides used by Bolivian farmers. Environ. Mol. Mutagen. 63, 4–17. 10.1002/em.22468 34881454

[B13] Berenbaum M. C. (1985). The expected effect of a combination of agents: the general solution. J. Theor. Biol. 114, 413–431. 10.1016/S0022-5193(85)80176-4 4021503

[B14] Bian Y. Wang Y. Liu F. Li X. Wang B. (2020). The stability of four organophosphorus insecticides in stored cucumber samples is affected by additives. Food Chem. 331, 127352. 10.1016/j.foodchem.2020.127352 32652343

[B15] Brown D. D. R. Molinaro A. M. Pearson B. J. (2018). The planarian TCF/LEF factor Smed-tcf1 is required for the regeneration of dorsal-lateral neuronal subtypes. Dev. Biol. 433, 374–383. 10.1016/J.YDBIO.2017.08.024 29291981

[B16] Buratti F. M. Testai E. (2005). Malathion detoxification by human hepatic carboxylesterases and its inhibition by isomalathion and other pesticides. J. Biochem. Mol. Toxicol. 19, 406–414. 10.1002/JBT.20106 16421896

[B17] Buratti F. M. Volpe M. T. Meneguz A. Vittozzi L. Testai E. (2003). CYP-specific bioactivation of four organophosphorothioate pesticides by human liver microsomes. Toxicol. Appl. Pharmacol. 186, 143–154. 10.1016/S0041-008X(02)00027-3 12620367

[B18] Buratti F. M. D'Aniello A. Volpe M. T. Meneguz A. Testai E. (2005). Malathion bioactivation in the human liver: the contribution of different cytochrome p450 isoforms. Drug Metabolism Dispos. Biol. Fate Chem. 33, 295–302. 10.1124/dmd.104.001693 15557345

[B19] Cebrià F. Nakazawa M. Mineta K. Ikeo K. Gojobori T. Agata K. (2002). Dissecting planarian central nervous system regeneration by the expression of neural-specific genes. Dev. Growth Differ. 44 (2), 135–146. 10.1046/j.1440-169x.2002.00629.x 11940100

[B20] Cedergreen N. (2014). Quantifying synergy: a systematic review of mixture toxicity studies within environmental toxicology. PloS One 9, e96580. 10.1371/journal.pone.0096580 24794244 PMC4008607

[B21] Cochet-Escartin O. Mickolajczk K. J. Collins E.-M. S. (2015). Scrunching: a novel escape gait in planarians. Phys. Biol. 12, 056010. 10.1088/1478-3975/12/5/056010 26356147

[B22] Cole T. B. Jansen K. Park S. Li W. F. Furlong C. E. Costa L. G. (2010). The toxicity of mixtures of specific organophosphate compounds is modulated by paraoxonase 1 status. Adv. Exp. Med. Biol. 660, 47–60. 10.1007/978-1-60761-350-3_6 20221870 PMC3035621

[B23] Collins E. M. S. Hessel E. V. S. Hughes S. (2024). How neurobehavior and brain development in alternative whole-organism models can contribute to prediction of developmental neurotoxicity. NeuroToxicology 102, 48–57. 10.1016/j.neuro.2024.03.005 38552718 PMC11139590

[B24] Costa L. G. (2018). Organophosphorus compounds at 80: some old and new issues. Toxicol. Sci. 162, 24–35. 10.1093/toxsci/kfx266 29228398 PMC6693380

[B25] Costa L. G. Richter R. J. Li W. F. Cole T. Guizzetti M. Furlong C. E. (2008). Paraoxonase (PON 1) as a biomarker of susceptibility for organophosphate toxicity. Biomarkers 8, 1–12. 10.1080/13547500210148315 12519632

[B26] Crow J. A. Bittles V. Herring K. L. Borazjani A. Potter P. M. Ross M. K. (2012). Inhibition of recombinant human carboxylesterase 1 and 2 and monoacylglycerol lipase by chlorpyrifos oxon, paraoxon and methyl paraoxon. Toxicol. Appl. Pharmacol. 258, 145–150. 10.1016/j.taap.2011.10.017 22100607 PMC3345137

[B27] Di Consiglio E. Pistollato F. Mendoza-De Gyves E. Bal-Price A. Testai E. (2020). Integrating biokinetics and in vitro studies to evaluate developmental neurotoxicity induced by chlorpyrifos in human iPSC-derived neural stem cells undergoing differentiation towards neuronal and glial cells. Reprod. Toxicol. 98, 174–188. 10.1016/j.reprotox.2020.09.010 33011216 PMC7772889

[B28] Dunkel J. Talbot J. Schötz E.-M. (2011). Memory and obesity affect the population dynamics of asexual freshwater planarians. Phys. Biology 8, 026003. 10.1088/1478-3975/8/2/026003 21263170

[B29] Ellman G. L. Courtney K. D. Andres V. Feather-Stone R. M. (1961). A new and rapid colorimetric determination of acetylcholinesterase activity. Biochem. Pharmacol. 7, 88–95. 10.1016/0006-2952(61)90145-9 13726518

[B30] French M. C. Hall C. Harrison J. Inch T. Sellers D. Smith A. (1985). Stereodependent bioactivation of R(+)-ethyl S-propyl methylphosphonothioate: is the S-oxide the active metabolite? Pesticide Biochem. Physiology 24, 53–60. 10.1016/0048-3575(85)90113-0

[B31] Fuselier S. G. Ireland D. Fu N. Rabeler C. Collins E. M. S. (2023). Comparative toxicity assessment of glyphosate and two commercial formulations in the planarian Dugesia japonica. Front. Toxicol. 5, 1–12. 10.3389/ftox.2023.1200881 37435546 PMC10332155

[B32] Geier M. C. James Minick D. Truong L. Tilton S. Pande P. Anderson K. A. (2018). Systematic developmental neurotoxicity assessment of a representative PAH Superfund mixture using zebrafish. Toxicol. Appl. Pharmacol. 354, 115–125. 10.1016/j.taap.2018.03.029 29630969 PMC6087484

[B33] Grube A. (2011). Pesticides industry sales and usage: 2006 and 2007 market estimates. U.S. Environmental Protection Agency, 1–41.

[B34] Hagstrom D. Cochet-Escartin O. Zhang S. Khuu C. Collins E. M. S. (2015). Freshwater planarians as an alternative animal model for neurotoxicology. Toxicol. Sci. 147, 270–285. 10.1093/toxsci/kfv129 26116028 PMC4838007

[B35] Hagstrom D. Hirokawa H. Zhang L. Radic Z. Taylor P. Collins E. M. S. (2017). Planarian cholinesterase: in vitro characterization of an evolutionarily ancient enzyme to study organophosphorus pesticide toxicity and reactivation. Archives Toxicol. 91, 2837–2847. 10.1007/s00204-016-1908-3 27990564 PMC6485937

[B36] Hagstrom D. Zhang S. Ho A. Tsai E. S. Radić Z. Jahromi A. (2018). Planarian cholinesterase: molecular and functional characterization of an evolutionarily ancient enzyme to study organophosphorus pesticide toxicity. Archives Toxicol. 92, 1161–1176. 10.1007/s00204-017-2130-7 29167930 PMC6413736

[B37] Hsieh J. H. Ryan K. Sedykh A. Lin J. A. Shapiro A. J. Parham F. (2019). Application of benchmark concentration (BMC) analysis on zebrafish data: a new perspective for quantifying toxicity in alternative animal models. Toxicol. Sci. 167, 282–292. 10.1093/toxsci/kfy258 30321397 PMC6317423

[B38] ICCVAM (Interagency Coordinating Committee on the Validation of Alternative Methods) (2018). A strategic roadmap for establishing new approaches to evaluate the safety of chemicals and medical products in the United States. ICCVAM. 10.22427/NTP-ICCVAM-ROADMAP2018

[B39] International Agency for Research on Cancer (2017). Some organophospate insecticides and herbicides, 112. Lyon, France: World Health Organization. 31829533

[B40] Ireland D. Collins E. S. (2023). Planarians as a model to study neurotoxic agents. In Alternative methods in neurotoxicology (Elsevier Inc.), 29–60. 10.1016/bs.ant.2023.01.002

[B41] Ireland D. Bochenek V. Chaiken D. Rabeler C. Onoe S. Soni A. (2020). Dugesia japonica is the best suited of three planarian species for high-throughput toxicology screening. Chemosphere 253, 126718. 10.1016/j.chemosphere.2020.126718 32298908 PMC7350771

[B42] Ireland D. Rabeler C. Gong T. Collins E.-M. S. (2022a). Bioactivation and detoxification of organophosphorus pesticides in freshwater planarians shares similarities with humans. Archives Toxicol. 96, 3233–3243. 10.1007/s00204-022-03387-y 36173421 PMC10729609

[B43] Ireland D. Zhang S. Bochenek V. Hsieh J.-H. Rabeler C. Meyer Z. (2022b). Differences in neurotoxic outcomes of organophosphorus pesticides revealed via multi-dimensional screening in adult and regenerating planarians. Front. Toxicol. 4, 948455. 10.3389/ftox.2022.948455 36267428 PMC9578561

[B44] Ireland D. Coffinas E. Rabeler C. Collins E.-M. S. (2025a). Planarian behavioral screening is a useful invertebrate model for evaluating seizurogenic chemicals. BioRxiv, 10.1101/2025.10.07.680963 41724271 PMC13037453

[B45] Ireland D. Word L. J. Collins E.-M. S. (2025b). Statistical analysis of multi-endpoint phenotypic screening increases sensitivity of planarian neurotoxicity testing. Toxicol. Sci. 208, 104–121. 10.1093/toxsci/kfaf117 40839344 PMC12404179

[B46] Jansen K. L. Cole T. B. Park S. S. Furlong C. E. Costa L. G. (2009). Paraoxonase 1 (PON1) modulates the toxicity of mixed organophosphorus compounds. Toxicol. Appl. Pharmacol. 236, 142–153. 10.1016/j.taap.2009.02.001 19371602 PMC2717945

[B47] Kao T.-S. Fukuto T. R. (1977). Metabolism of O,S-dimethyl propionyl- and hexanoylphosphoramidothioate in the house fly and white mouse. Pesticide Biochem. Physiology 7 (1), 83–95. 10.1016/0048-3575(77)90069-4

[B48] Karanth S. Olivier K. Liu J. Pope C. (2001). *In* vivo interaction between chlorpyrifos and parathion in adult rats: sequence of administration can markedly influence toxic outcome. Toxicol. Applied Pharmacology 177, 247–255. 10.1006/taap.2001.9312 11749124

[B49] Khan H. A. A. Akram W. Shad S. A. Lee J. J. (2013). Insecticide mixtures could enhance the toxicity of insecticides in a resistant dairy population of *Musca domestica* L. PLoS ONE 8, e60929. 10.1371/journal.pone.0060929 23613758 PMC3628707

[B50] Kortenkamp A. Backhaus T. Faust M. (2009). State of the Art Report on Mixture Toxicity - Final report, executive summary. Technical Report 10293409. Brussels, Belgium: European Commission.

[B51] Laetz C. A. Baldwin D. H. Collier T. K. Hebert V. Stark J. D. Scholz N. L. (2009). The synergistic toxicity of pesticide mixtures: implications for risk assessment and the conservation of endangered Pacific salmon. Environ. Health Perspect. 117, 348–353. 10.1289/ehp.0800096 19337507 PMC2661902

[B52] Leonel Javeres M. N. Raza S. Judith N. Anwar F. Habib R. Batool S. (2020). Mixture of organophosphates chronic exposure and pancreatic dysregulations in two different population samples. Front. Public Health 8, 534902. 10.3389/fpubh.2020.534902 33194944 PMC7655777

[B53] Liu L. Liu S. S. Yu M. Zhang J. Chen F. (2015). Concentration addition prediction for a multiple-component mixture containing no effect chemicals. Anal. Methods 7, 9912–9917. 10.1039/C5AY01784J

[B54] Loewe S. (1928). Die quantitativen Probleme der Pharmakologie. Ergeb. Physiol. 27, 47–187. 10.1007/BF02322290

[B55] Madgwick P. G. Kanitz R. (2023). Beyond redundant kill: a fundamental explanation of how insecticide mixtures work for resistance management. Pest Manag. Sci. 79, 495–506. 10.1002/ps.7180 36098048 PMC10092901

[B56] Main A. R. Dauterman W. C. (1967). Kinetics for the inhibition of carboxylesterase by malaoxon. Can. J. Biochem. 45, 757–771. 10.1139/o67-087 6034697

[B57] Martin T. Ochou O. G. Vaissayre M. Fournier D. (2003). Organophosphorus insecticides synergize pyrethroids in the resistant strain of cotton bollworm, Helicoverpa armigera (Hübner) (Lepidoptera: noctuidae) from West Africa. J. Econ. Entomology 96 (2), 468–474. 10.1093/jee/96.2.468 14994817

[B58] Martin O. Scholze M. Ermler S. McPhie J. Bopp S. K. Kienzler A. (2021). Ten years of research on synergisms and antagonisms in chemical mixtures: a systematic review and quantitative reappraisal of mixture studies. Environ. Int. 146, 106206. 10.1016/j.envint.2020.106206 33120228

[B59] Meigs L. Smirnova L. Rovida C. Leist M. Hartung T. (2018). Animal testing and its alternatives – the most important omics is economics. ALTEX 35, 275–305. 10.14573/altex.1807041 30008008

[B60] Metcalfe M. (2002). The economic importance of organophosphates in California agriculture.

[B61] Mileson B. E. Chambers J. E. Chen W. L. Dettbarn W. Ehrich M. Eldefrawi A. T. (1998). Common mechanism of toxicity: a case study of organophosphorus pesticides. Toxicol. Sci. 41, 8–20. 10.1006/toxs.1997.2431 9520337

[B62] Moser V. C. Casey M. Hamm A. Carter W. H. Simmons J. E. Gennings C. (2005). Neurotoxicological and statistical analyses of a mixture of five organophosphorus pesticides using a ray design. Toxicol. Sci. 86 (1), 101–115. 10.1093/toxsci/kfi163 15800032

[B63] Murphy S. D. Dubois K. P. (1957). Quantitative measurement of inhibition of the enzymatic detoxification of Malathion by EPN (ethyl p-nitrophenyl thionobenzenephosphonate). Exp. Biol. Med. 96, 813–818. 10.3181/00379727-96-23617 13505868

[B64] Murphy S. D. Anderson R. L. DuBois K. P. (1959). Potentiation of toxicity of malathion by triorthotolyl phosphate. Exp. Biol. Med. 100, 483–487. 10.3181/00379727-100-24668 13634179

[B65] National Pesticide Information Center (2009). Diazinon technical fact sheet. Corvallis, OR: Oregon State University.

[B66] Nillos M. G. Rodriguez-Fuentes G. Gan J. Schlenk D. (2007). Enantioselective acetylcholinesterase inhibition of the organophosphorous insecticides profenofos, fonofos, and crotoxyphos. Environ. Toxicol. Chem. 26, 1949–1954. 10.1897/07-001R.1 17705656

[B67] Office of Pesticide Programs, U.S. Environmental Protection Agency (EPA) (2000). The use of data on cholinesterase inhibition in risk assessments of organophosphorous and carbamate pesticides. Washington, D.C.: US Environmental Protection Agency.

[B68] Ojha A. Srivastava N. (2014). *In* vitro studies on organophosphate pesticides induced oxidative DNA damage in rat lymphocytes. Mutat. Res. Genet. Toxicol. Environ. Mutagen. 761, 10–17. 10.1016/j.mrgentox.2014.01.007 24468856

[B69] Osuma E. A. Riggs D. W. Gibb A. A. Hill B. G. (2018). High throughput measurement of metabolism in planarians reveals activation of glycolysis during regeneration. Regeneration 5, 78–86. 10.1002/REG2.95 29721328 PMC5911454

[B70] Pacis J. Ireland D. Coffinas E. Sheehan J. Sun K. Collins E. M. S. (2025). Red midge larvae are an invertebrate alternative diet to beef liver for planarian husbandry. Biol. Life Sci. 10.20944/preprints202511.0502.v1 PMC12730930 41463316

[B71] Peiris T. H. Hoyer K. K. Oviedo N. J. (2014). Innate immune system and tissue regeneration in planarians: an area ripe for exploration. Seminars Immunol. 26, 295–302. 10.1016/j.smim.2014.06.005 25082737 PMC4171206

[B72] Peter J. V. Sudarsan T. I. Moran J. L. (2014). Clinical features of organophosphate poisoning: a review of different classification systems and approaches. Indian J. Crit. Care Med. 18, 735–745. 10.4103/0972-5229.144017 25425841 PMC4238091

[B73] Poet T. S. Wu H. Kousba A. A. Timchalk C. (2003). *In* vitro rat hepatic and intestinal metabolism of the organophosphate pesticides chlorpyrifos and diazinon. Toxicol. Sciences An Official Journal Soc. Toxicol. 72, 193–200. 10.1093/TOXSCI/KFG035 12655035

[B74] Pope C. Karanth S. Liu J. (2005). Pharmacology and toxicology of cholinesterase inhibitors: uses and misuses of a common mechanism of action. Environ. Toxicology Pharmacology 19, 433–446. 10.1016/j.etap.2004.12.048 21783509

[B75] Qu W. Crizer D. M. DeVito M. J. Waidyanatha S. Xia M. Houck K. (2021). Exploration of xenobiotic metabolism within cell lines used for Tox21 chemical screening. Toxicol. Vitro An International Journal Published Association BIBRA 73, 105109. 10.1016/j.tiv.2021.105109 33609632 PMC10838150

[B76] R Core Team (2021). R: a Language and environment for statistical computing. Vienna, Austria: R Foundation for Statistical Computing.

[B77] Richardson J. R. Chambers H. W. Chambers J. E. (2001). Analysis of the additivity of in Vitro inhibition of cholinesterase by mixtures of Chlorpyrifos-oxon and Azinphos-methyl-oxon. Toxicol. Appl. Pharmacol. 172, 128–139. 10.1006/taap.2001.9140 11298499

[B78] Ritz C. Baty F. Streibig J. C. Gerhard D. (2015). Dose-response analysis using R. PLOS ONE 10, e0146021. 10.1371/JOURNAL.PONE.0146021 26717316 PMC4696819

[B79] Ritz C. Streibig J. C. Kniss A. (2021). How to use statistics to claim antagonism and synergism from binary mixture experiments. Pest Manag. Sci. 77, 3890–3899. 10.1002/ps.6348 33644956

[B80] Ross K. G. Currie K. W. Pearson B. J. Zayas R. M. (2017). Nervous system development and regeneration in freshwater planarians. Wiley Interdiscip. Rev. Dev. Biol. 6, 1–26. 10.1002/wdev.266 28326682

[B81] Russom C. L. LaLone C. A. Villeneuve D. L. Ankley G. T. (2014). Development of an adverse outcome pathway for acetylcholinesterase inhibition leading to acute mortality. Environ. Toxicology Chemistry 33 (10), 2157–2169. 10.1002/etc.2662 24922588

[B82] Schopfer L. M. Tacal O. Masson P. Lockridge O. (2025). Kinetic analysis suggests that malathion at low environmental exposures is not toxic to humans. PLOS One 20, e0335361. 10.1371/journal.pone.0335361 41134844 PMC12551860

[B83] Silva M. Pham N. Lewis C. Iyer S. Kwok E. Solomon G. (2015). A comparison of ToxCast test results with in vivo and other in vitro endpoints for neuro, endocrine, and developmental toxicities: a case study using endosulfan and methidathion. Birth Defects Res. B Dev. Reproductive Toxicol. 104, 71–89. 10.1002/BDRB.21140 26017137

[B84] Srikanth N. S. Seth P. K. (1990). Alterations in xenobiotic metabolizing enzymes in brain and liver of rats coexposed to endosulfan and malathion. J. Appl. Toxicol. 10, 157–160. 10.1002/jat.2550100303 2380476

[B85] Sultana Shaik A. Shaik A. P. Jamil K. Alsaeed A. H. (2016). Evaluation of cytotoxicity and genotoxicity of pesticide mixtures on lymphocytes. Toxicol. Mech. Methods 26, 588–594. 10.1080/15376516.2016.1218577 27603568

[B86] Taylor (2018). Anticholinesterase agents. In Goodman and Gilman’s the pharmacological basis of therapeutics. Editor Brunton L. L. (San Francisco: McGraw Hill Education), 163–176.

[B87] U.S. EPA (2003). Advances in dose addition for chemical mixtures: a white paper. EPA/100/R-23/001. Washington, DC: U.S. Environmental Protection Agency.

[B88] US Federal Drug Administration (2025). Roadmap to reducing animal testing in preclinical safety studies. In Federal drug administration.

[B89] USEPA (2021). EPA new approach methods work plan (v2). Washington, DC: U.S. Environmental Protection Agency.

[B90] Wang P. Jiang S. Liu D. Zhang H. Zhou Z. (2006). Enantiomeric resolution of chiral pesticides by high-performance liquid chromatography. J. Agric. Food Chem. 54, 1577–1583. 10.1021/jf052631o 16506803

[B91] Wang Y. Liu S. S. Huang P. Wang Z. J. Xu Y. Q. (2021). Assessing the combined toxicity of carbamate mixtures as well as organophosphorus mixtures to Caenorhabditis elegans using the locomotion behaviors as endpoints. Sci. Total Environ. 760, 143378. 10.1016/j.scitotenv.2020.143378 33168241

[B92] Wiesner J. Kriz Z. Kuca K. Jun D. Koca J. (2007). Acetylcholinesterases – the structural similarities and differences. J. Enzyme Inhibition Med. Chem. 22 (4), 417–424. 10.1080/14756360701421294 17847707

[B93] Wing K. D. Glickman A. H. Casida J. E. (1983). Oxidative bioactivation of S-alkyl phosphorothiolate pesticides: stereospecificity of profenofos insecticide activation. Science 219, 63–65. 10.1126/science.6849116 6849116

[B94] Woodruff T. J. (2003). America’s children and the environment: measures of contaminants, body burdens, and illnesses. Second Edition. U.S. Environmental Protection Agency.

[B95] Wu X. Yang X. Majumder A. Swetenburg R. Goodfellow F. T. Bartlett M. G. (2017). From the cover: astrocytes are protective against chlorpyrifos developmental neurotoxicity in human pluripotent stem cell-derived astrocyte-neuron cocultures. Toxicol. Sci. 157, 410–420. 10.1093/toxsci/kfx056 28369648

[B96] Yamada S. Kubo Y. Yamazaki D. Sekino Y. Kanda Y. (2017). Chlorpyrifos inhibits neural induction via Mfn1-mediated mitochondrial dysfunction in human induced pluripotent stem cells. Sci. Rep. 7, 40925. 10.1038/srep40925 28112198 PMC5256306

[B97] Yan J. Xiang B. Wang D. Tang S. Teng M. Yan S. (2019). Different toxic effects of racemate, enantiomers, and metabolite of malathion on HepG2 cells using high-performance liquid chromatography-quadrupole-time-of-flight-based metabolomics. J. Agric. Food Chem. 67, 1784–1794. 10.1021/acs.jafc.8b04536 30673264

[B98] Zhang G. Truong L. Tanguay R. L. Reif D. M. (2017). A new statistical approach to characterize chemical-elicited behavioral effects in high-throughput studies using zebrafish. PLOS ONE 12, e0169408. 10.1371/journal.pone.0169408 28099482 PMC5242475

[B99] Zhang S. Hagstrom D. Hayes P. Graham A. Collins E.-M. S. (2019a). Multi-behavioral endpoint testing of an 87-chemical compound library in freshwater planarians. Toxicol. Sci. 167, 26–44. 10.1093/toxsci/kfy145 29893936 PMC6657585

[B100] Zhang S. Ireland D. Sipes N. S. Behl M. Collins E.-M. S. (2019b). Screening for neurotoxic potential of 15 flame retardants using freshwater planarians. Neurotoxicology Teratology 73, 54–66. 10.1016/j.ntt.2019.03.003 30943442 PMC9524722

