# Supplementary material for: Rapid behavioral screening in the planarian Dugesia japonica is a biologically relevant system to study neurotoxicity of organophosphorus pesticides mixtures
Source: Front Toxicol. 2026 Mar 26;8:1753546. doi: 10.3389/ftox.2026.1753546 (PMC13061384; doi:10.3389/ftox.2026.1753546)
Supplement: Supplementary file 1 [file DataSheet4.pdf]

Supplementary Material

1 Supplementary Data

Supplementary File 1. Mass spectroscopy results to independently confirm chemical purity. .zip files containing reports from Lotus Separations details methods and results of high performance liquid chromatography / mass spectroscopy analysis.

Supplementary File 2. Benchmark concentration (BMC), confidence intervals and hit scores for all readouts in adult and regenerating planarians exposed to OP mixtures. The BMC is listed as BMC_median. BMC_cil and BMC_ciu represent the lower and upper confidence intervals, respectively. Hit_confidence scores greater than 0.5 were considered hits. Some readouts were assessed in both increasing (+) and decreasing (-) directions and are listed separately. Concentrations are listed in log(M).

Supplementary File 3. Benchmark concentration (BMC), confidence intervals and hit scores for all readouts in adult and regenerating planarians exposed to individual OPs. The BMC is listed as BMC_median. BMC_cil and BMC_ciu represent the lower and upper confidence intervals, respectively. Hit_confidence scores greater than 0.5 were considered hits. Some readouts were assessed in both increating (+) and decreasing (-) directions and are listed separately. Concentrations are listed in log(M). Some readouts were previously reported in (Ireland *et al.*, 2022).

2 Supplementary Figures and Tables

2.1 Supplementary Figures

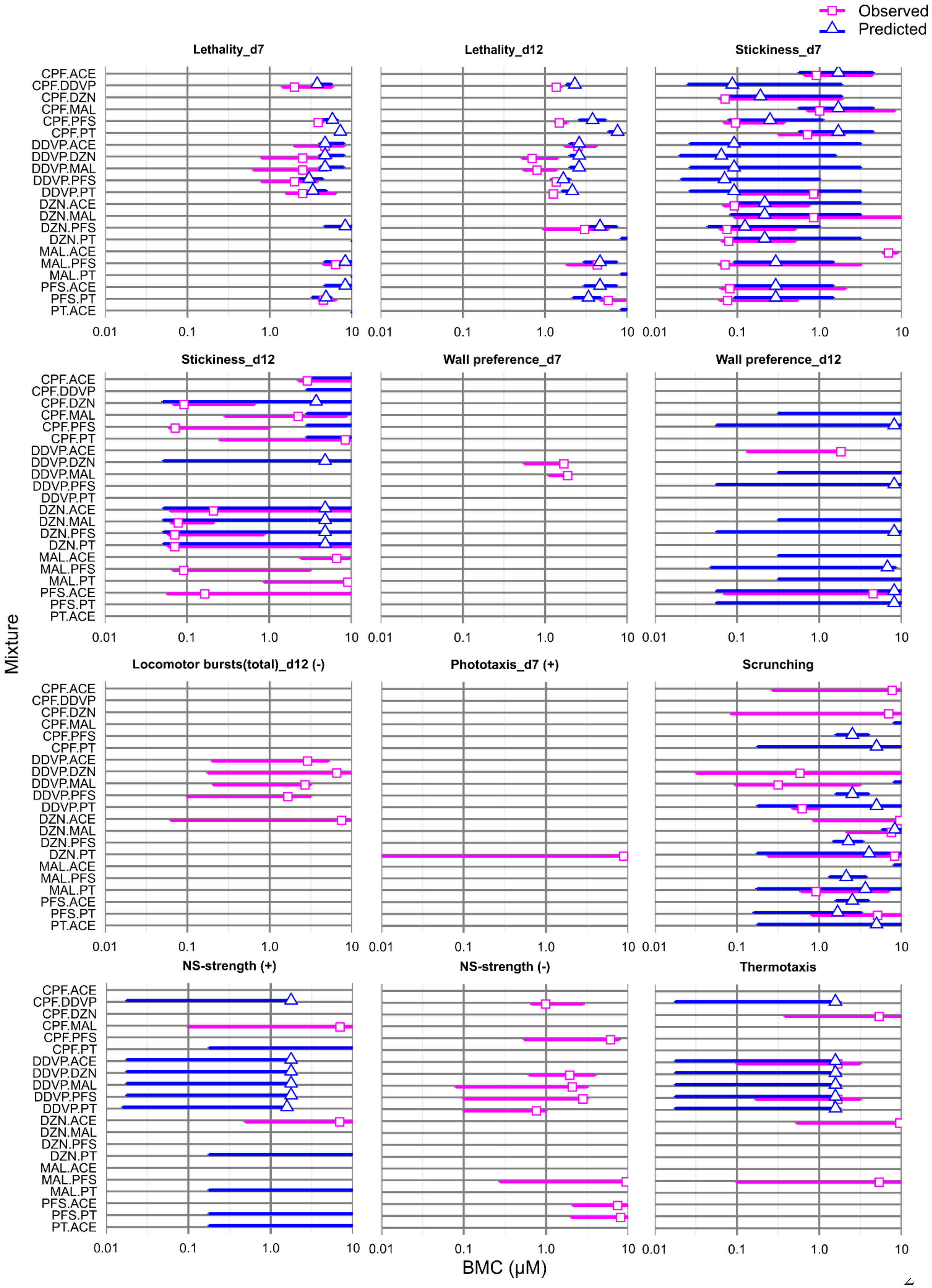
Observed
Predicted
Lethality_d7
Lethality_d12
Stickiness_d7
Stickiness_d12
Wall preference_d7
Wall preference_d12
Locomotor bursts(total)_d12 (-)
Phototaxis_d7 (+)
Scrunching
NS-strength (+)
NS-strength (-)
Thermotaxis
CPF.ACE
CPF.DDVP
CPF.DZN
CPF.MAL
CPF.PFS
CPF.PT
DDVP.ACE
DDVP.DZN
DDVP.MAL
DDVP.PFS
DDVP.PT
DZN.ACE
DZN.MAL
DZN.PFS
DZN.PT
MAL.ACE
MAL.PFS
MAL.PT
PFS.ACE
PFS.PT
PT.ACE
0.01
0.1
1.0
10
Mixture
BMC (µM)

Supplementary Figure 1. Comparison of BMCs predicted by CA model vs experimental BMCs of OP mixtures in adult planarians for non-locomotor endpoints. Observed refers to experimental mixture data; Predicted is predicted by CA model using single OP BMC data as input. Only predicted values up to 10 µM are considered as this was the maximum concentration tested in the experimental mixtures. Markers indicate median BMC, error bars represent 95% confidence intervals.

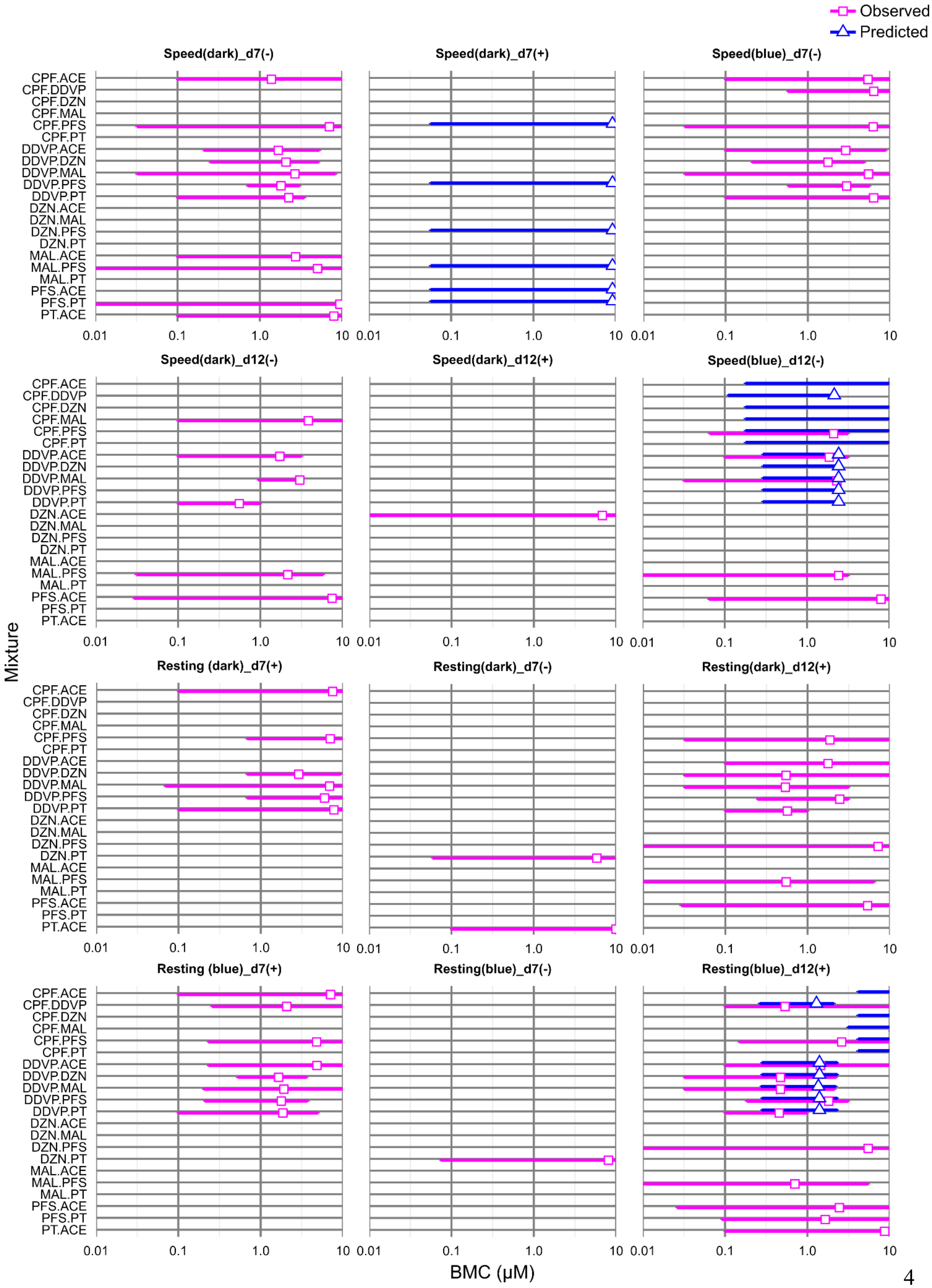
Observed
Predicted
Speed(dark)_d7(-)
Speed(dark)_d7(+)
Speed(blue)_d7(-)
Speed(dark)_d12(-)
Speed(dark)_d12(+)
Speed(blue)_d12(-)
Resting (dark)_d7(+)
Resting(dark)_d7(-)
Resting(dark)_d12(+)
Resting (blue)_d7(+)
Resting(blue)_d7(-)
Resting(blue)_d12(+)
CPF.ACE
CPF.DDVP
CPF.DZN
CPF.MAL
CPF.PFS
CPF.PT
DDVP.ACE
DDVP.DZN
DDVP.MAL
DDVP.PFS
DDVP.PT
DZN.ACE
DZN.MAL
DZN.PFS
DZN.PT
MAL.ACE
MAL.PFS
MAL.PT
PFS.ACE
PFS.PT
PT.ACE
Mixture
0.01
0.1
1.0
10
BMC (μM)

Supplementary Figure 2. Comparison of BMCs predicted by CA model vs experimental BMCs of OP mixtures in adult planarians for locomotor (speed and resting) endpoints. Observed refers to experimental mixture data; Predicted is predicted by CA model using single OP BMC data as input. Only predicted values up to 10 μM are considered as this was the maximum concentration tested in the experimental mixtures. Markers indicate median BMC, error bars represent 95% confidence intervals.

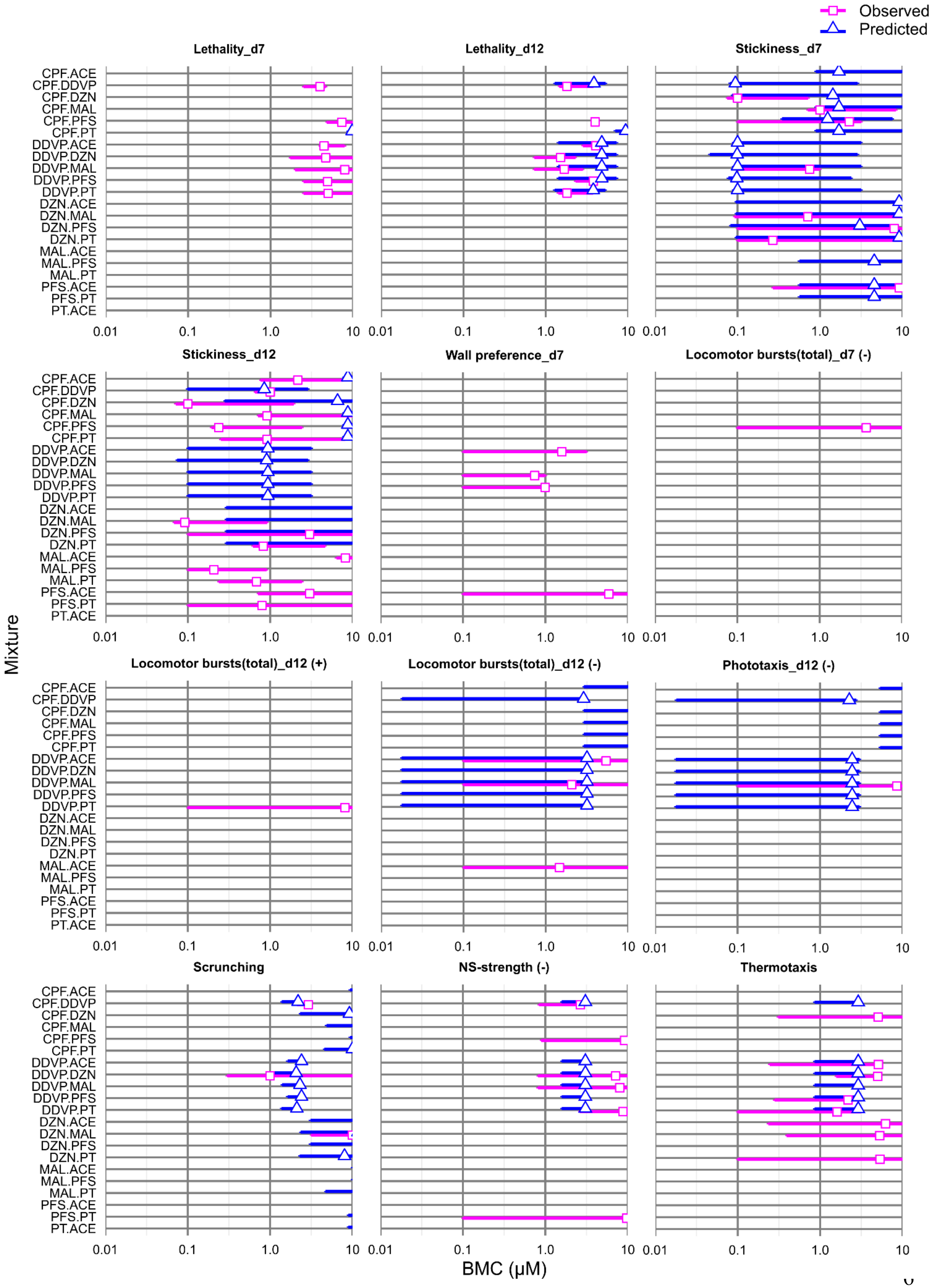
Observed
Predicted
Lethality_d7
Lethality_d12
Stickiness_d7
Stickiness_d12
Wall preference_d7
Locomotor bursts(total)_d7 (-)
Locomotor bursts(total)_d12 (+)
Locomotor bursts(total)_d12 (-)
Phototaxis_d12 (-)
Scrunching
NS-strength (-)
Thermotaxis
CPF.ACE
CPF.DDVP
CPF.DZN
CPF.MAL
CPF.PFS
CPF.PT
DDVP.ACE
DDVP.DZN
DDVP.MAL
DDVP.PFS
DDVP.PT
DZN.ACE
DZN.MAL
DZN.PFS
DZN.PT
MAL.ACE
MAL.PFS
MAL.PT
PFS.ACE
PFS.PT
PT.ACE
0.01
0.1
1.0
10
Mixture
BMC (µM)

Supplementary Figure 3. Comparison of BMCs predicted by CA model vs experimental BMCs of OP mixtures in regenerating planarians for non-locomotor endpoints. Observed refers to experimental mixture data; Predicted is predicted by CA model using single OP BMC data as input. Only predicted values up to 10 μM are considered as this was the maximum concentration tested in the experimental mixtures. Markers indicate median BMC, error bars represent 95% confidence intervals.

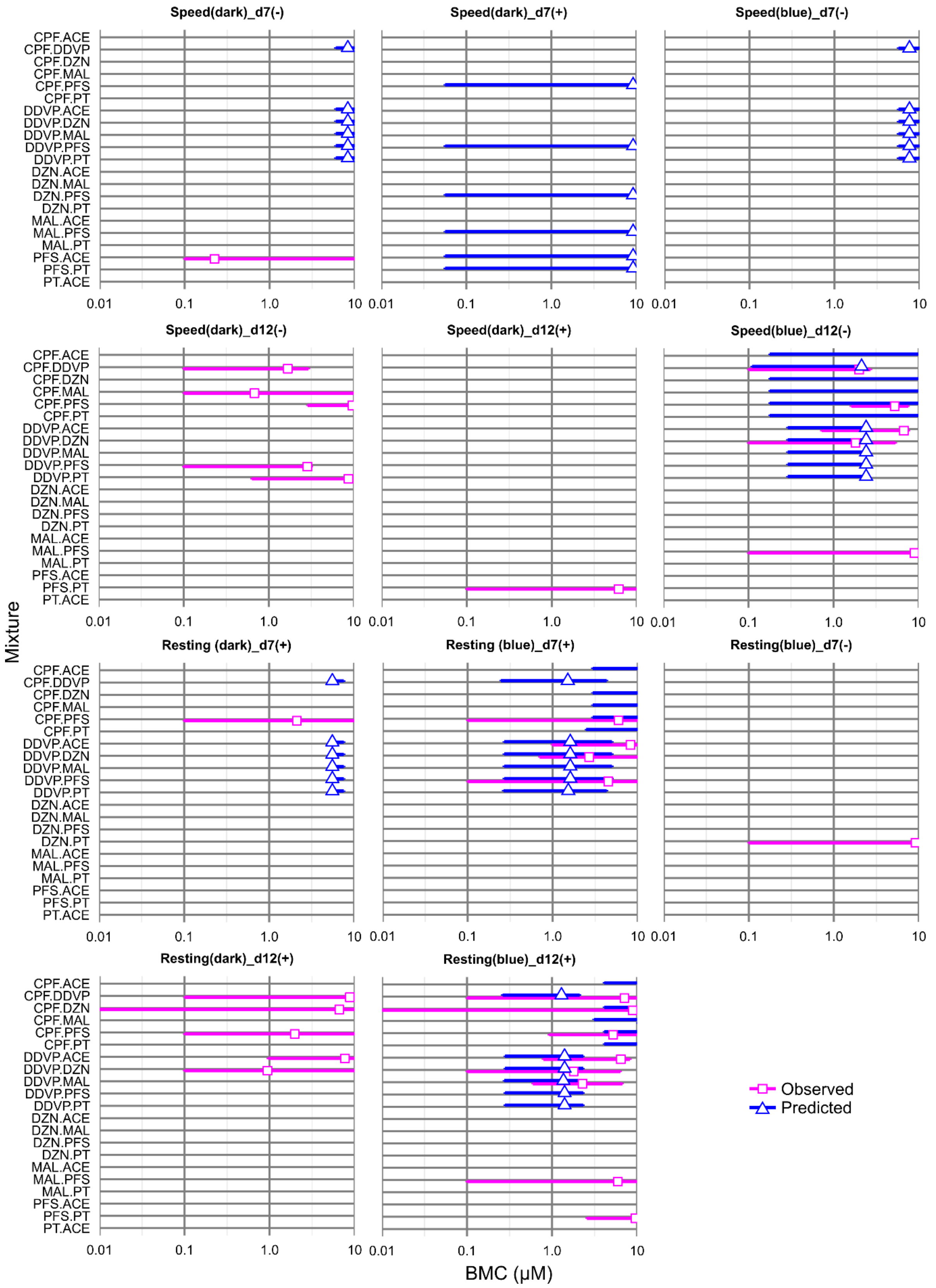
Speed(dark)_d7(-)
Speed(dark)_d7(+)
Speed(blue)_d7(-)
Speed(dark)_d12(-)
Speed(dark)_d12(+)
Speed(blue)_d12(-)
Resting (dark)_d7(+)
Resting (blue)_d7(+)
Resting(blue)_d7(-)
Resting(dark)_d12(+)
Resting(blue)_d12(+)
CPF.ACE
CPF.DDVP
CPF.DZN
CPF.MAL
CPF.PFS
CPF.PT
DDVP.ACE
DDVP.DZN
DDVP.MAL
DDVP.PFS
DDVP.PT
DZN.ACE
DZN.MAL
DZN.PFS
DZN.PT
MAL.ACE
MAL.PFS
MAL.PT
PFS.ACE
PFS.PT
PT.ACE
0.01
0.1
1.0
10
Mixture
BMC (µM)
Observed
Predicted

Supplementary Figure 4. Comparison of BMCs predicted by CA model vs experimental BMCs of OP mixtures in regenerating planarians for locomotor (speed and resting) endpoints. Observed refers to experimental mixture data; Predicted is predicted by CA model using single OP BMC data as input. Only predicted values up to 10 μM are considered as this was the maximum concentration tested in the experimental mixtures. Markers indicate median BMC, error bars represent 95% confidence intervals.

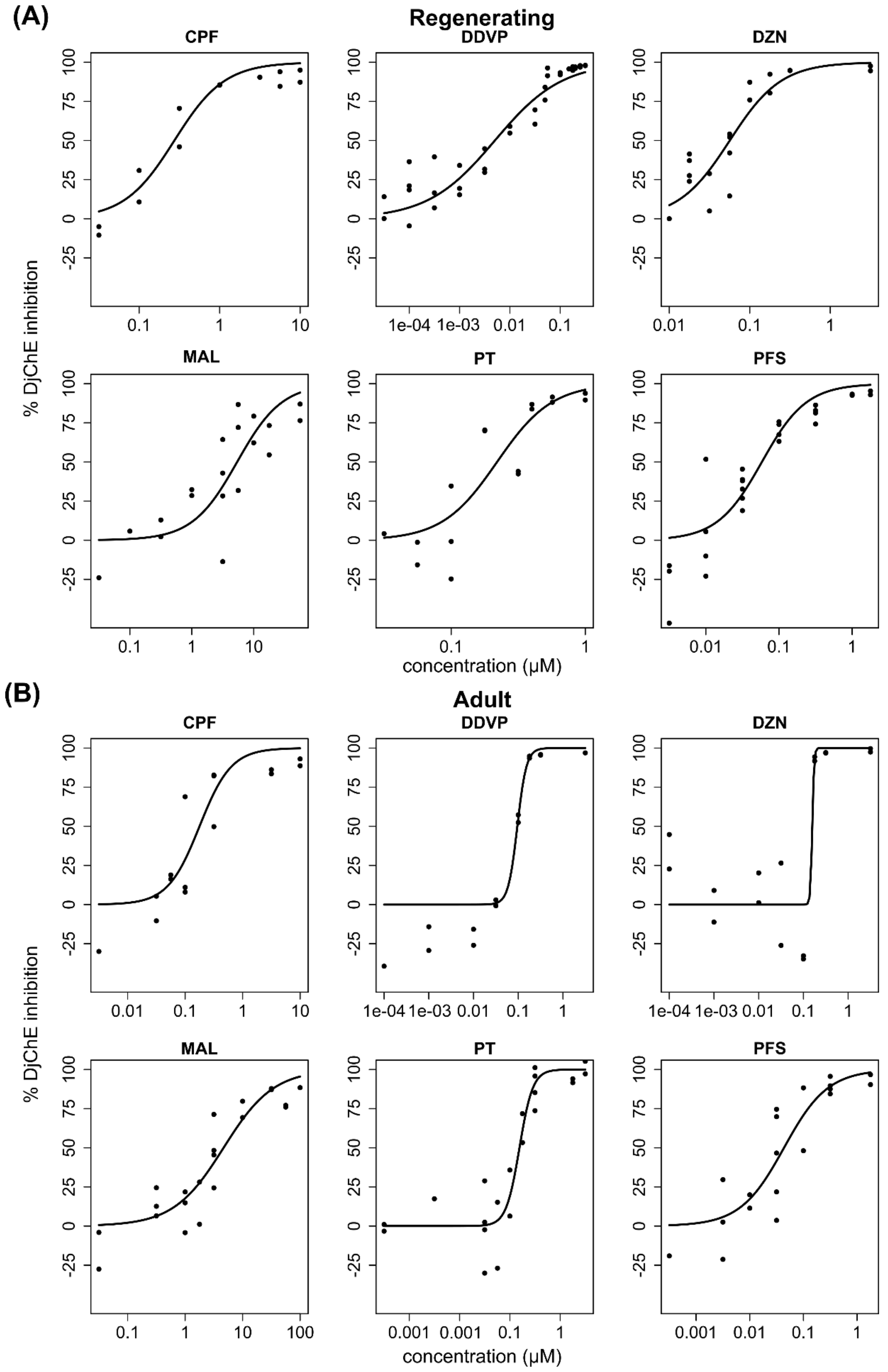

Supplementary Figure 5. AChE inhibition in response to single OP exposure. Ellman assays were performed on (A) regenerating and (B) adult planarians exposed for 12 days to different concentrations of the

OPs. Dose response curves were fit with a Hill equation (setting the lower limit to 0 and the upper limit to 100) using the R package drc.

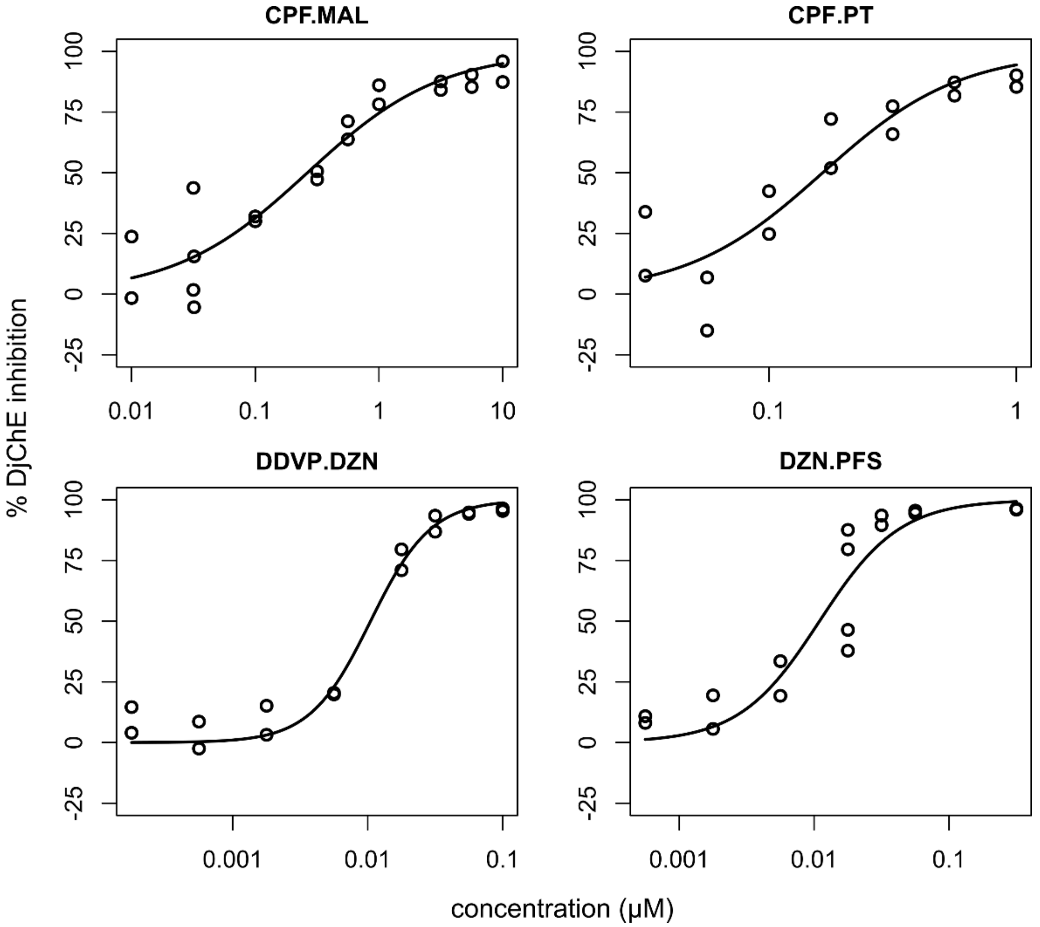

Supplementary Figure 6. AChE inhibition in response to select binary OP mixture exposure in regenerating planarians. Ellman assays were performed on regenerating planarians exposed for 12 days to different concentrations of the listed OP mixtures. Mixtures were equimolar concentrations of the two OPs and concentrations reflect the concentrations of the component OPs. Dose response curves were fit with a Hill equation (setting the lower limit to 0 and the upper limit to 100) using the R package drc.

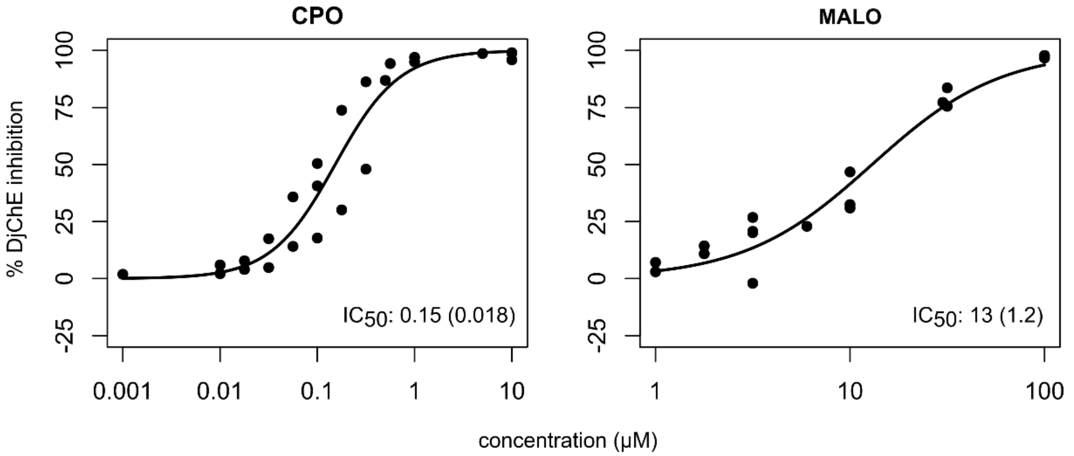

Supplementary Figure 7. AChE inhibition *in vitro* for chlorpyrifos oxon (CPO) and malaoxon (MALO) following 10-minute treatment. Ellman assays were performed on adult planarian homogenates exposed for 10 minutes to different concentrations of CPO and MALO. Dose response curves were fit with a Hill equation (setting the lower limit to 0 and the upper limit to 100) using the R package drc. The 10 minute IC_{50} along with standard error in parentheses is listed for each oxon.

2.2 Supplementary Tables

Supplementary Table 1. Mixtures run at lower concentrations

Mixture	Additional test concentrations (µM)	Worm type
CPF.DZN	0.01, 0.0316	Adult/Regenerating
CPF.PFS	0.0316	Adult
DZN.MAL	0.01, 0.0316	Adult/Regenerating
DDVP.DZN	0.0316	Adult
DZN.PT	0.01, 0.0316	Adult
DZN.PFS	0.01, 0.0316	Adult
DDVP.MAL	0.0316	Adult
DZN.ACE	0.01, 0.0316	Adult
MAL.PFS	0.01, 0.0316	Adult
PFS.ACE	0.01, 0.0316	Adult
PFS.PT	0.01, 0.0316	Adult

Supplementary Table 2. Concentrations of OPs tested in Ellman assays

Chemical or Mixture	Concentration (µM)	Developmental stage	Experiment type
Chlorpyrifos<br>(CPF)	0.00316 -10<br>0.0316 - 10	Adult[1]<br>Regenerating	*In vivo*
Dichlorvos<br>(DDVP)	0.0001 - 3.16<br>0.0000316 - 0.316	Adult[1]<br>Regenerating	*In vivo*
Diazinon<br>(DZN)	0.0001-3.16<br>0.01-3.16	Adult[1]<br>Regenerating	*In vivo*
Malathion<br>(MAL)	0.0316-100<br>0.0316-56.2	Adult[1]<br>Regenerating	*In vivo*
Parathion<br>(PT)	0.000316-3.16<br>0.0316-1	Adult[1]<br>Regenerating	*In vivo*
Profenofos (PFS)	0.000316-1.78<br>0.00316-1.78	Adult[1]<br>Regenerating	*In vivo*
Acephate (ACE)	316	Adult/Regenerating	*In vivo*
CPF.MAL	0.01 – 10	Regenerating	*In vivo*
CPF.PT	0.0316 – 1	Regenerating	*In vivo*
DDVP.DZN	0.000178 - 0.1	Regenerating	*In vivo*
DZN.PFS	0.00056 - 0.316	Regenerating	*In vivo*
Chlorpyrifos Oxon<br>(CPO)	0.00001 - 10	Adult	*In vitro*
Malaoxon (MALO)	1- 100	Adult	*In vitro*

[1]Some of the adult data was previously published in (Ireland *et al.*, 2022)

Supplementary Table 3. Concentrations of CPO and MALO in mixture for *in vitro* Ellman assays. All concentrations are listed in µM. The ratio of [CPO]/[MALO] was held constant at 0.007 based on preliminary analysis of the IC_{50} values from the single oxon data.

**Ratio x IC_{50}**	**[CPO]**	**[MALO]**
8	0.8	108.8
4	0.4	54.4
2	0.2	27.2
1	0.1	13.6
1/2	0.05	6.8
1/4	0.025	3.4
1/8	0.0125	1.7
1/16	0.00625	0.85
