# Supplementary material for: Rapid behavioral screening in the planarian Dugesia japonica is a biologically relevant system to study neurotoxicity of organophosphorus pesticides mixtures
Source: Front Toxicol. 2026 Mar 26;8:1753546. doi: 10.3389/ftox.2026.1753546 (PMC13061384; doi:10.3389/ftox.2026.1753546)

Lotus Separations

Final Report

Chemical Purity & Integrity Analysis

**Type of Study:**	LC/UV/MS Purity and Integrity Analysis	**Sample ID:**	10
**Requestor:**	Eva-Maria Collin	**Email:**	ecollin3@swarthmore.edu
**Company:**	Swarthmore College	**Date:**	13-Mar-2014

Summary

The sample was received for purity and integrity analysis. A HPLC/UV/MS method was developed to ensure that the peak of interest was resolved from all impurities detectable by MS and UV detectors and no coelution was evidenced based on the LC/UV peak homogeneity test. The LC/UV peak area percent purity was determined to be **99.5% at 220 to 380 nm** wavelength window with two impurities of 0.4% and 0.1%.

The integrity of the sample was confirmed by LC/MS of the major peak based on the observed molecular ion in the positive and negative ion modes. The LC/UV peak homogeneity was examined by comparing the UV spectra across the chromatographic peak, and no co-elution was evidenced.

The chromatograph conditions are described in the Experimental Details section. The representative chromatograms, mass and UV spectra, peak homogeneity and purity results are enclosed in the report.

Experimental Details

Analytical HPLC-UV-MS Conditions

Instrument:	Agilent 1200 HPLC/MS
Column:	Agilent Poroshell 120 EC-C18, 2.7 µm 3.0 (ID) x 100 (L) mm
Temperature:	40˚C
Mobile Phase:	A: 0.1% formic acid in water; B: 0.1% formic acid in acetonitrile
	Gradient: 5% B (0 min); 98% B (10 min); 95% B (13 min)
Flow rate:	0.5 mL/min
UV Wavelength:	220-380 nm
MS ionization mode:	ESI positive and negative ion modes
Mass range:	200 – 800
Sample diluent:	Water/MeOH/MeCN

Chemical Purity & Integrity Analysis

Analytical LC/MS Characterization Data:

Analytical chromatograms the sample (top) and solvent blank (bottom) at 220 to 380 nm:

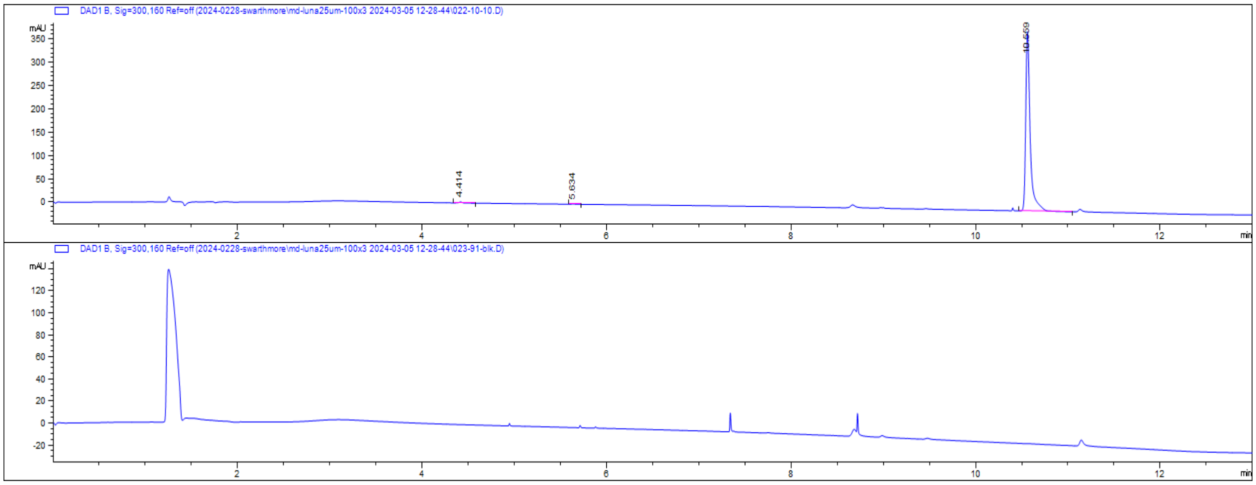

Integrated peak area% results:

#	Time	Type	Area	Height	Width	Area%
1	4.414	BB	5.2	2.2	0.0355	0.402
2	5.634	BB	1.9	6.8E-1	0.0418	0.147
3	10.559	BB	1297.8	383.8	0.0499	99.451

Mass Spectrum in positive ion mode of the major component:

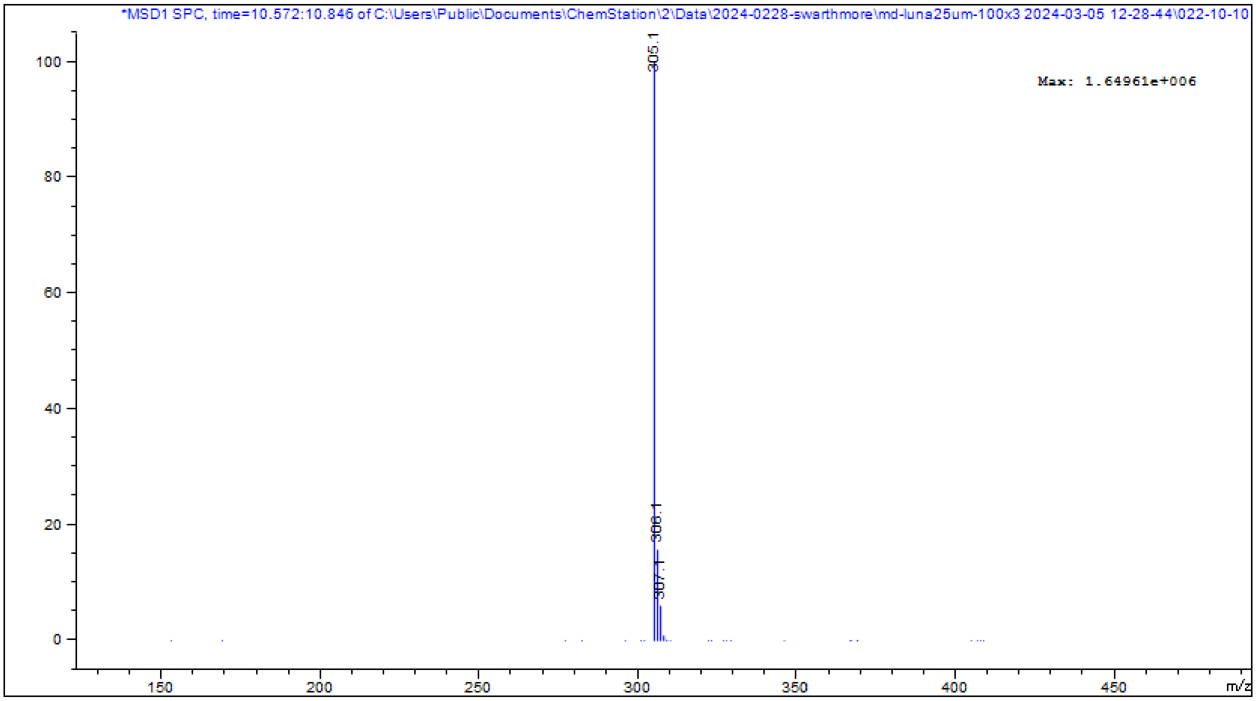

Chemical Purity & Integrity Analysis

Overlay of seven UV spectra across the LC/UV peak of interest demonstrating the peak homogeneity:

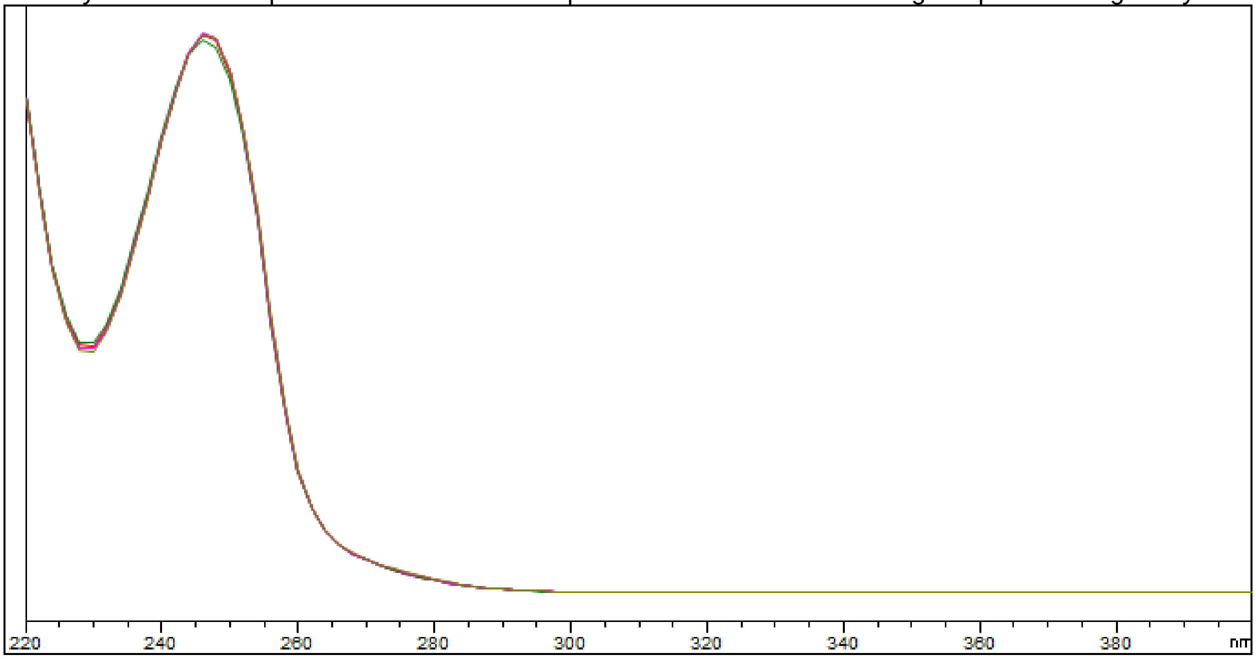

Spectral overlay of the major component and the impurities:

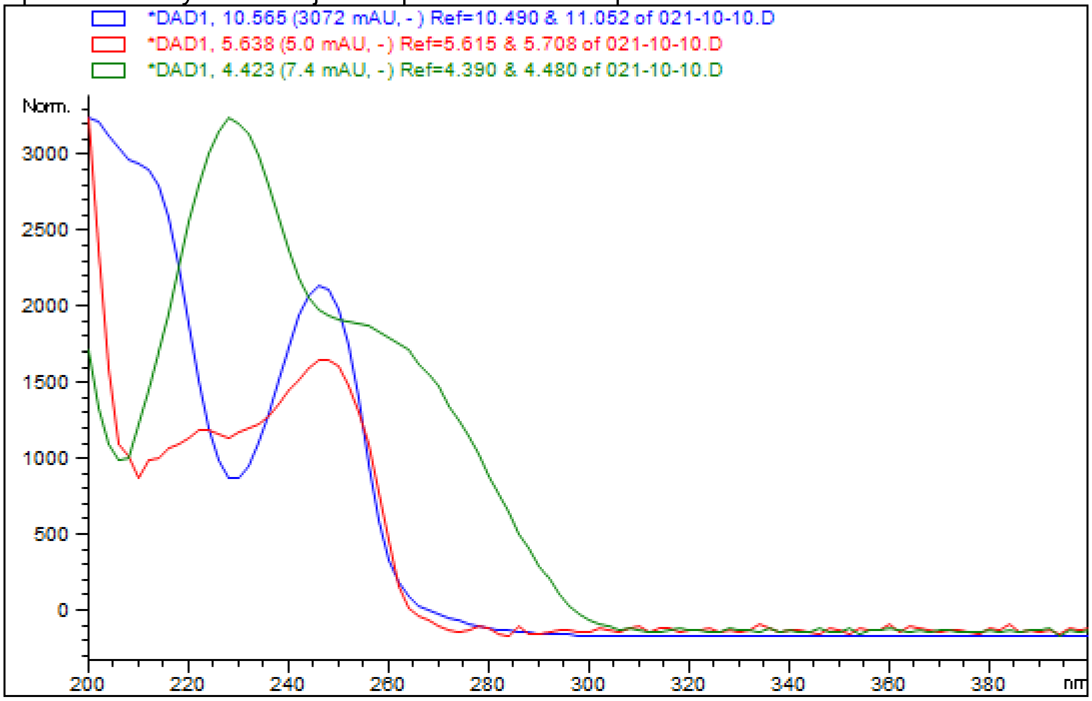

Supplement: Supplementary file 2 [file DataSheet1.zip › Mass spec/Diazinon_LC-MS purity and integrity.pdf]
