# Supplementary material for: Rapid behavioral screening in the planarian Dugesia japonica is a biologically relevant system to study neurotoxicity of organophosphorus pesticides mixtures
Source: Front Toxicol. 2026 Mar 26;8:1753546. doi: 10.3389/ftox.2026.1753546 (PMC13061384; doi:10.3389/ftox.2026.1753546)

Final Report

Chemical Purity & Integrity Analysis

**Type of Study:**	LC/UV/MS Purity and Integrity Analysis	**Sample ID:**	15
**Requestor:**	Maury Hutchens	**Email:**	Mhutche1@swarthmore.edu
**Company:**	Swarthmore College	**Date:**	01-May-2014

Summary

The sample was received for purity and integrity analysis. A HPLC/UV/MS method was developed, and no impurity was detected. The LC/UV peak area percent purity was determined to be **greater than 99.9% at 220 nm**.

The integrity of the sample was confirmed by LC/MS of the major peak based on the observed molecular ion in the positive ion mode. The LC/UV peak homogeneity was examined by comparing the UV spectra across the chromatographic peak, and no co-elution was evidenced.

The chromatograph conditions are described in the Experimental Details section. The representative chromatograms, mass and UV spectra, peak homogeneity and purity results are enclosed in the report.

Experimental Details

Analytical HPLC-UV-MS Conditions

Instrument:	Agilent 1200 HPLC/MS
Column:	Phenomenex, Omega Polar C18 3.5 µm 3.0 (ID) x 150 (L) mm
Temperature:	40˚C
Mobile Phase:	A: 0.1% formic acid in water; B: 0.1% formic acid in acetonitrile<br>Gradient: 5% B (0 min); 98% B (15 min); 98% B (18 min)
Flow rate:	0.5 mL/min
UV Wavelength:	220-380 nm
MS ionization mode:	ESI positive and negative ion modes
Mass range:	120 – 800 m/z
Sample diluent:	Water/EtOH

Final Report

Chemical Purity & Integrity Analysis

Analytical LC/MS Characterization Data:

Analytical chromatograms the sample at 220 nm:

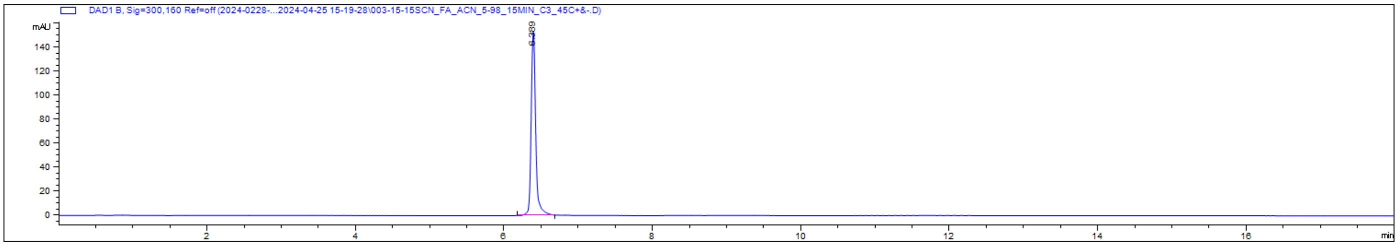

Integrated peak area% results:

#	Time	Type	Area	Height	Width	Area%
1	6.713	BB	10873.6	1750.3	0.0945	100.000

Mass Spectrum in positive ion mode of the major component:

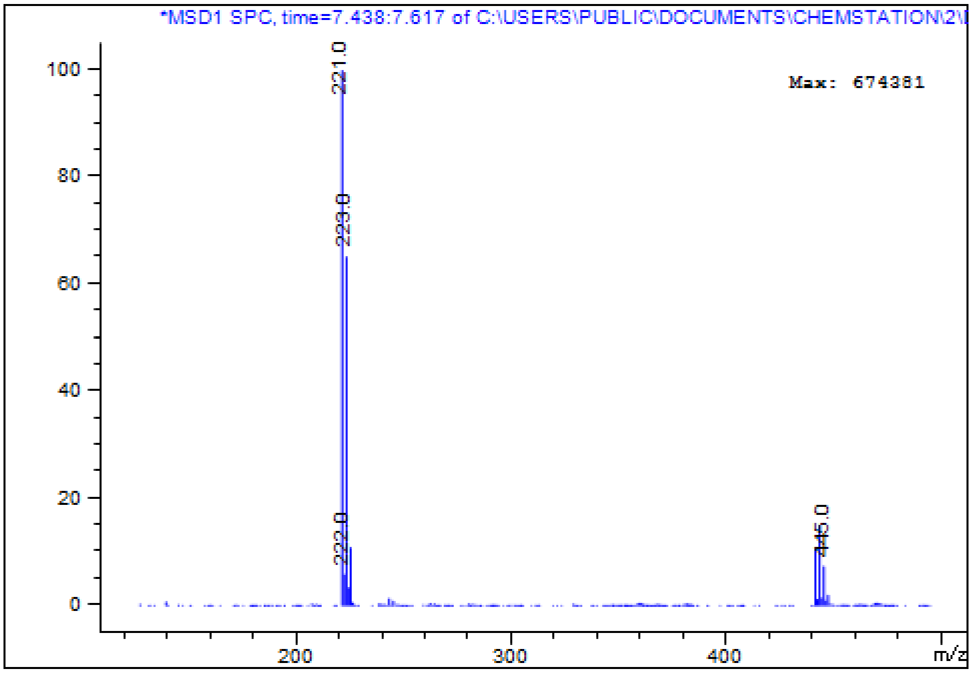

Chemical Purity & Integrity Analysis

Overlay of seven UV spectra across the LC/UV peak of interest demonstrating the peak homogeneity:

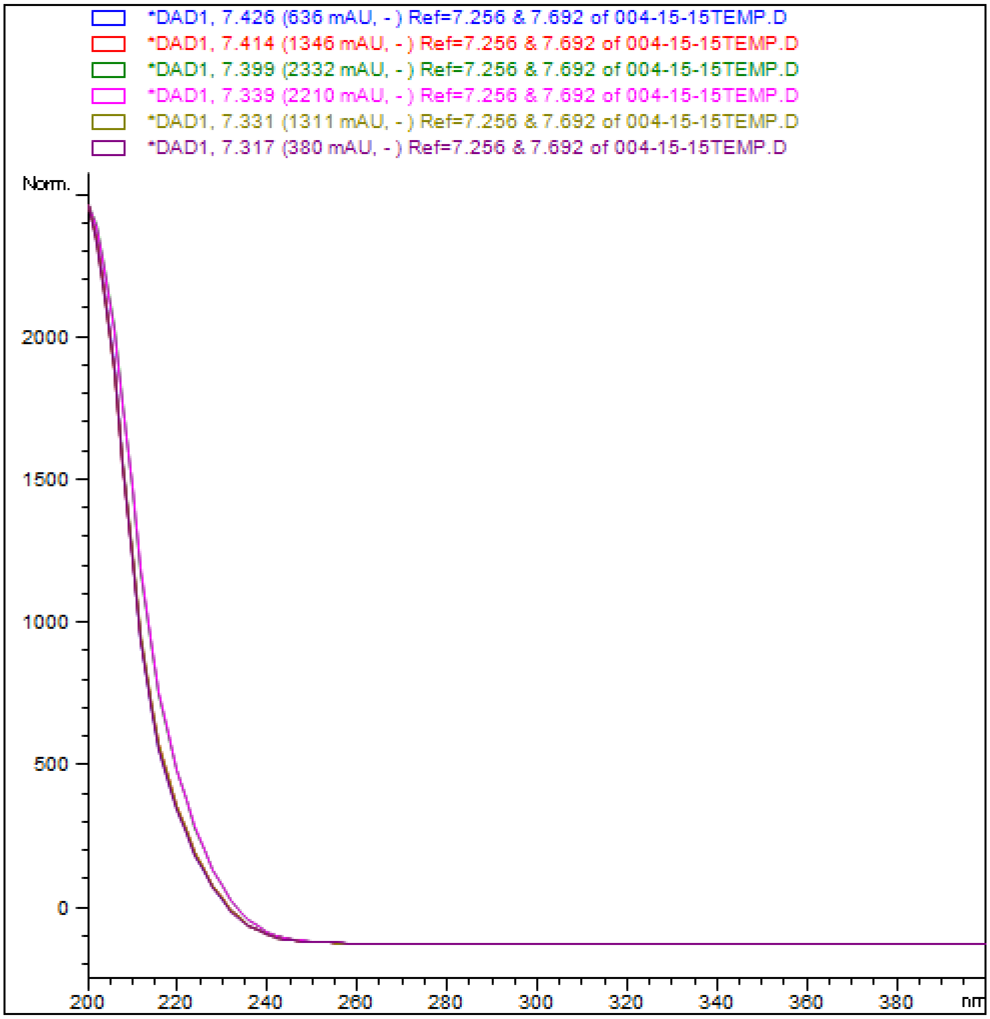

Supplement: Supplementary file 2 [file DataSheet1.zip › Mass spec/Dichlorvos_LC-MS purity and integrity.pdf]
